# Quantitation of Multiple Injection Dynamic PET Scans: An Investigation of the Benefits of Pooling Data from Separate Scans when Mapping Kinetics

**DOI:** 10.1088/1361-6560/ac0683

**Published:** 2021-07-01

**Authors:** Fengyun Gu, Finbarr O’Sullivan, Mark Muzi, David A. Mankoff

**Affiliations:** 1 Department of Statistics, University College Cork, Cork, Ireland; 2 Department of Radiology, University of Washington, Seattle, Washington, USA; 3 Department of Radiology, University of Pennsylvania, Philadelphia, USA

**Keywords:** Dynamic PET, Non-Parametric Residue Mapping, H_2_O-FDG Dual-Tracer Study, Repeat H_2_O Study, Combined Kinetic Analysis, FBP and ML Reconstructions

## Abstract

Multiple injection dynamic positron emission tomography (PET) scanning is used in the clinical management of certain groups of patients and in medical research. The analysis of these studies can be approached in two ways: (i) separate analysis of data from individual tracer injections, or (ii), concatenate/pool data from separate injections and carry out a combined analysis. The simplicity of separate analysis has some practical appeal but may not be statistically efficient. We use a linear model framework associated with a kinetic mapping scheme to develop a simplified theoretical understanding of separate and combined analysis. The theoretical framework is explored numerically using both 1-D and 2-D simulation models. These studies are motivated by the breast cancer flow-metabolism mismatch studies involving ^15^O-Water (H_2_O) and ^18^F-Fluorodeoxyglucose (FDG) and repeat ^15^O-H_2_O injections used in brain activation investigations. Numerical results are found to be substantially in line with the simple theoretical analysis: mean square error (MSE) characteristics of alternative methods are well described by factors involving the local voxel-level resolution of the imaging data, the relative activities of the individual scans and the number of separate injections involved. While voxel-level resolution has dependence on scan dose, after adjustment for this effect, the impact of a combined analysis is understood in simple terms associated with the linear model used for kinetic mapping. This is true for both data reconstructed by direct filtered backprojection (FBP) or iterative maximum likelihood (ML). The proposed analysis has potential to be applied to the emerging long axial field-of-view PET scanners.

## 1. Introduction

Positron emission tomography (PET) scanning is an important diagnostic imaging technique used in the management of patients with cancer and other diseases, as well as in medical research. In clinical settings, PET is typically used to create a single 3-D *static* image of the distribution of the tracer atoms in the field of view in a time-frame of interest, after the tracer has been administered. But PET can also be used to obtain a temporal sequence of scans after radiotracer injection. Such *dynamic* PET scans give the possibility to analyze the transport and retention of tracer in order to recover more detailed metabolic information (Cunningham and Jones; 1993; O’Sullivan; 1993). Most dynamic PET studies involve the use of a single radiotracer, such as ^15^O-Water (H_2_O) for blood flow (Herscovitch et al.; 1983), ^18^F-Fluorodeoxyglucose (FDG) for glucose metabolism (Phelps et al.; 1979) or ^11^C-verapamil for P-glycoprotein (P-gp) activity (Deo et al.; 2014). Studies in which multiple radiotracers are used in the same subject in the same imaging session have the potential to provide more comprehensive profiles of tissue function (Eary et al.; 2011; Kadrmas et al.; 2013), *in-vivo*. Examples include: repeat ^15^O-H_2_O imaging for brain activation studies (Beason-Held et al.; 1999), ^15^O-H_2_O and ^18^F-FDG for breast cancer (Mankoff et al.; 2002), ^13^N-NH3 and ^18^F-FDG for myocardial viability (Yamagishi et al.; 1999; Dilsizian et al.; 2013), ^15^O-H_2_O and ^11^C-Verapamil for assessment of multi-drug resistance (Eyal et al.; 2009).

While data from multiple radiotracer scans could be assembled *post-hoc* from separate imaging sessions, acquiring the data in a single imaging session has several advantages. (i) It can reduce issues of anatomic co-registration. This is important for comparing the facets of biology related to multiple tracers, especially for targets that may move between studies, for example, breast (Fowler et al.; 2016) and ovarian (Makvandi et al.; 2018) cancers comparing FDG PET/CT staging studies to therapeutic biomarkers. No doubt longer separation of injections can lead to more patient motions. (ii) It can reduce potential variation in the physiologic state of the subject from one session to the next (Huang et al.; 1982). This is especially true for metabolic studies using H_2_O and FDG or Glutamine and FDG (Zhou et al.; 2017). (iii) Tracer parameter estimation for multi-tracers may provide more reliable kinetic analysis due to common dependence of parameters: tracer delivery on flow or tracer clearance on plasma volume and hydration, etc.

With the development of new PET scanners like PennPET Explorer (Karp et al.; 2020) and Explorer total-body PET scanner (Badawi et al.; 2019), the longer field of view, increased temporal and spatial resolution, with lower dose are possible. These new scanners offer new opportunities for multi-tracer studies like the dual-tracer imaging with two 18F-labeled tracers using PennPET Explorer (Viswanath et al.; 2021; Viswanath; 2020) where combined data with conjoint modelling can help make more robust the comparison of regional differences in the relationship between the biologic parameters measures (Paquette et al.; 2020; Mankoff et al.; 2019).

**Figure 1:**
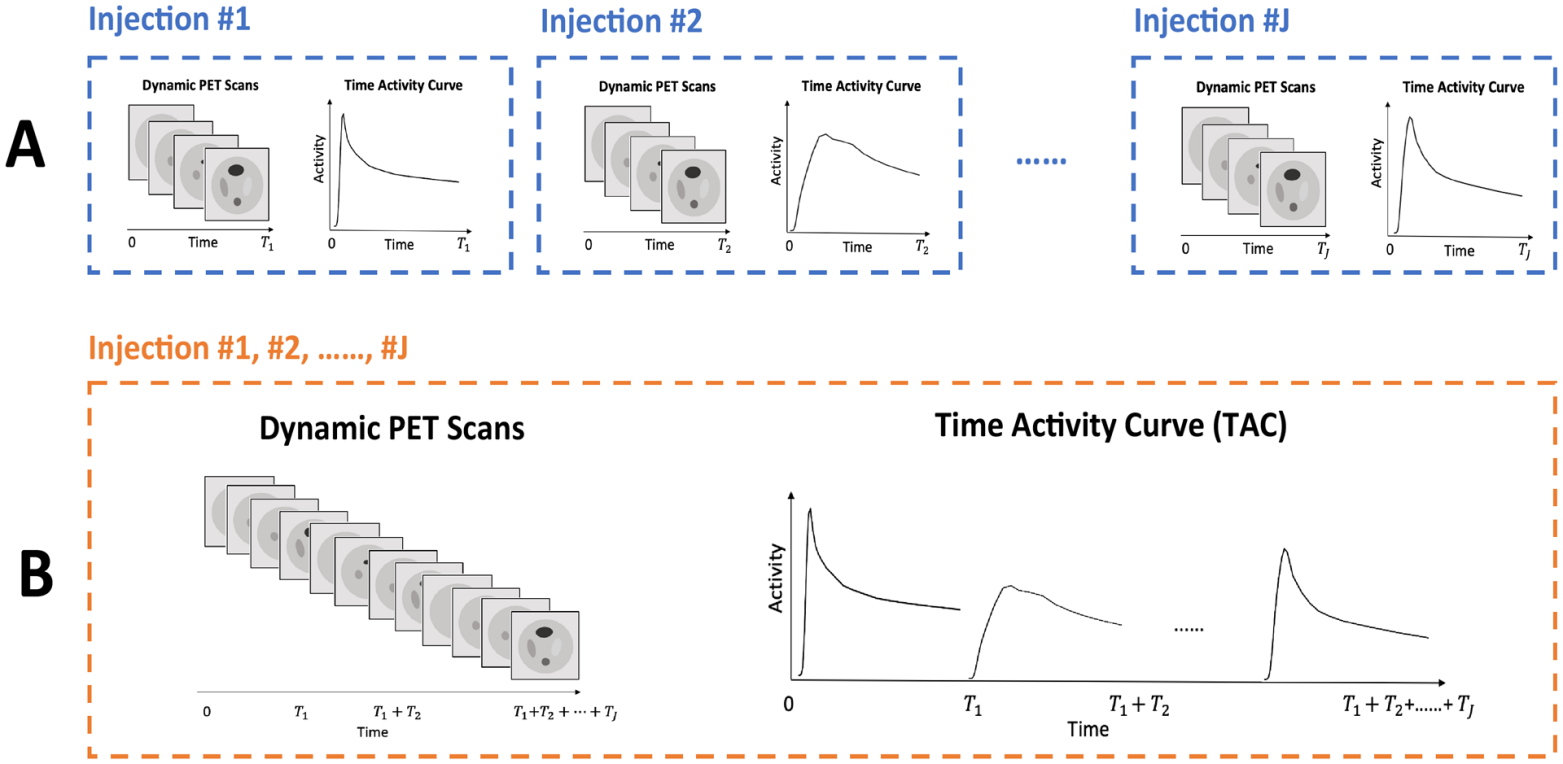
Multi-Injection Dynamic PET Studies. **A:** Separate Analysis; **B:** Combined Analysis.

Multi-tracer studies bring a range of significant challenges (Krohn et al.; 2007), data analysis being one. There are many situations in which the analysis of multi-tracer dynamic studies *must* consider the full temporal time-course of data, see for example, (Spence et al.; 1998; Rust and Kadrmas; 2005; Zhang et al.; 2016). However, whenever the temporal separation between different tracer injections is much longer than the radiotracer half-life or there is a simple mechanism to correct for spillover (Huang et al.; 1982; Koeppe et al.; 1998, 2001; Black et al.; 2009; El Fakhri et al.; 2013), analysis can be approached in one of two ways: (i) studies can be processed separately to recover kinetic information corresponding to individual tracers (Figure 1A), or (ii), studies can be concatenated temporally and a combined analysis (Figure 1B) of the resulting data carried out.

This paper develops a simplified theoretical framework for understanding the relative performance of separate and combined analysis of multi-tracer PET studies. The approach is based on a type of linear modelling framework for mapping kinetics (O’Sullivan; 1993; O’Sullivan et al.; 2014) - details in section 2. The theoretical analysis suggests that after controlling for voxel-level imaging accuracy (resolution), the relative performance of separate and combine kinetic mapping can be understood in simplified terms. A series of numerical experiments, motivated by flow-metabolism mismatch and repeat brain activation studies, are used to directly assess mean square error (MSE) characteristics of combined and separate analysis techniques. These studies consider both filtered backprojection (FBP) and maximum likelihood (ML) reconstructed data as well as a number of important factors including, overall dose, the relative dose of individual tracers and the number of injections involved. Data from numerical experiments are subjected to careful statistical analysis. This helps to clarify the main findings. In flow-metabolism studies, combined analysis improves MSE performance by between 8% and 30%, depending on the reconstruction method used. In the repeat injection studies, MSE improvements increase linearly with the number of injections involved. This is in line with the proposed theory.

The outline of the paper is as follows: The kinetic mapping approach and the associated analysis of the impact of data combination are developed in section 2. An illustrative practical example from a flow-metabolism mismatch study in a breast cancer patient is given in section 3. Section 4 describes a series of numerical studies based on 1-D and 2-D scanning models. Results are presented in section 5. Section 6 presents a detailed statistical analysis of simulation results. The paper concludes with discussion.

## 2. Theory

**Figure 2:**
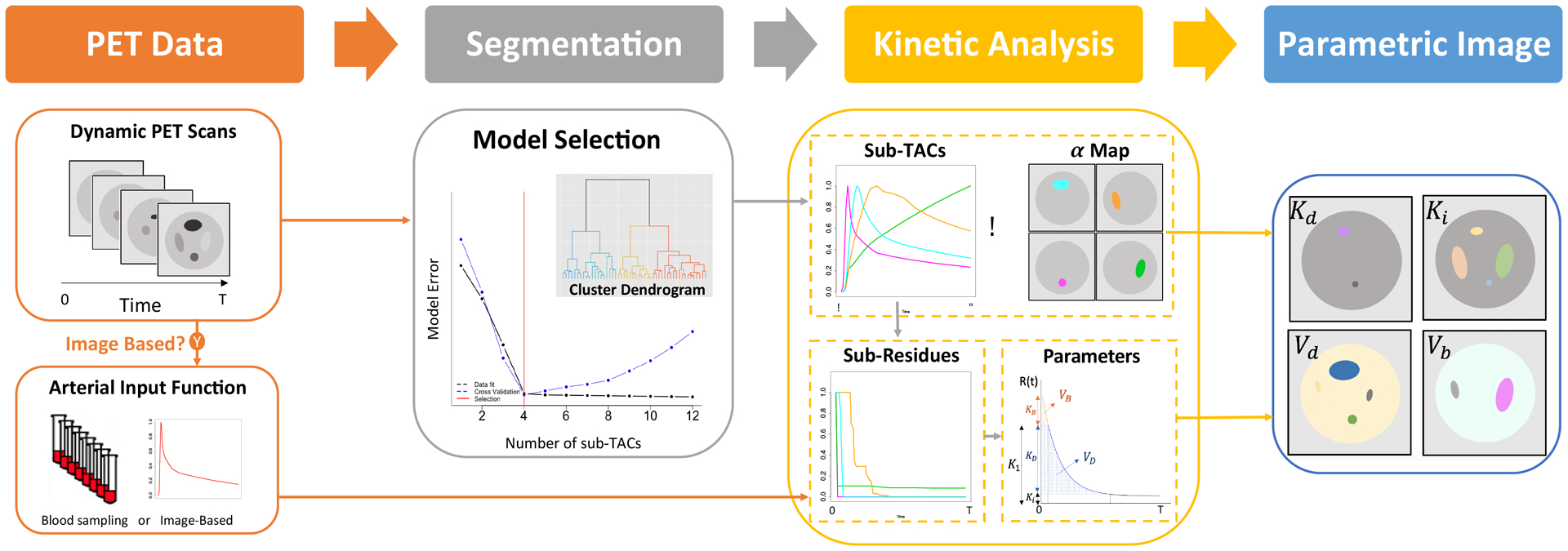
Non-parametric residue mapping (NPRM) process.

The focus is on dynamic PET studies in which it can be assumed that the tracer’s interaction with tissue is substantially linear and time-invariant. This assumption is valid for a large class of tracers used in PET, particularly in a cancer setting. The interpretation of time-course data from dynamic studies can be approached using classical indicator dilution theory (Meier and Zierler; 1954). This theory expresses the measured time-course as a convolution between the arterial input function (AIF) and the so-called tissue residue or impulse response function. The tissue residue is a necessarily monotone decreasing function. Widely used compartmental models (Kety and Schmidt; 1948; Phelps et al.; 1979), typically approximate the residue as a positive linear combination of 1 or 2 mono-exponential functions. The spectral method (Cunningham and Jones; 1993) generalizes this to allow the positive combination of potentially many mono-exponentials. In statistical terms, the residue may be viewed as a life-table for tracer atoms introduced to the tissue at time zero via the arterial supply - indeed this is a key part of (Meier and Zierler; 1954). As life-tables can be represented in terms of the underlying distribution (density/histogram) of travel times associated with the physiologic/metabolic journey of individual tracer atoms in the tissue, any residue modelling assumption carries with it a specification for the distribution of such travel times within the volume of tissue being analyzed (O’Sullivan et al.; 2009). Compartmental and spectral approximation requires that travel-time densities be spiked at time zero and be monotone decreasing. However, strict monotonicity of travel-time densities may not always be reasonable (Li et al.; 1997). One might imagine this may only be practically important in studies with high-frequency temporal sampling - perhaps only in the context of the emerging PET scanning technologies (Badawi et al.; 2019; Karp et al.; 2020). However (O’Sullivan et al.; 2009) used circa-1996 PET scanner data involving relatively crude temporal sampling to demonstrate significant statistical deficiencies in the ability of the standard 2-compartmental model to correctly represent the regional cerebral time-course of PET-FDG data in highly homogeneous brain regions of normal subjects. The anomaly was identified by considering a more flexible non-parametric analysis of the residue function, with suitable cross-validation adjustment for potential overfitting relative to the 2-compartmental model. In light of this finding, we have been motivated to employ a non-parametric representation of the tissue residue, particularly when analyzing PET time-course data corresponding to large and potentially heterogeneous regions of interest. The concept has been incorporated into a non-parametric residue mapping (NPRM) methodology that we have found useful for parametric imaging of dynamic data from PET imaging of cancer (O’Sullivan; 1993; O’Sullivan et al.; 2014). Figure 2 shows a schematic. Before we consider the topic of combined analysis of multiple-injection studies, we elaborate on important details of the NPRM approach. Theoretical points are illustrated numerically in the context of kinetic mapping PET-FDG data.

### 2.1. Voxel-level Kinetic Mapping by the NPRM Approach

There is extensive literature on techniques for mapping kinetics from dynamic PET data - see (Wang et al.; 2020) for a recent review including many references. A broad family of techniques, including (Cunningham and Jones; 1993; O’Sullivan; 1993), involves voxel-level representation of the tissue residue as a (positive) linear combination of suitable basis functions. Mono-exponential basis residues are used in (Cunningham and Jones; 1993); more elaborate basis elements, incorporating potentially complex blood-tissue exchange models are proposed in (O’Sullivan; 1993). NPRM technique is a variation on the latter approach in which basis elements (known as sub-TACs) are represented in terms of flexible model-free residues. A full account of the method with several cancer imaging applications is provided in (O’Sullivan et al.; 2014). In the current implementation of NPRM, sub-TACs are constructed by a **2-step** data-adaptive process. We elaborate on these steps and indicate how derived kinetic are mapped.

**Step 1** applies a standard recursive hierarchical clustering scheme to partition the image volume into a set of *S* segments or clusters, with the property that the time course data for voxels in the same segment are each approximately proportional to the mean time-course for that segment. Typically the number of segments needed for this is on the order of 100–200. The mean PET-measured time-course data for segments (${\bar{z}}_{s}$) together with their standard deviations (${\bar{\sigma }}_{s}$) are used to produce a reduced data set $D_{S}=\left\{\left({\bar{z}}_{sl},{\bar{\sigma }}_{sl}\right),s=1,2,\ldots ,S,l=1,2,\ldots ,N_{T}\right\}$. Here *N*_*T*_ is the number of time-frame of PET data acquisition. Next, the mean time-course data for each segment is modelled to create an associated set of modelled elements $M=\left\{{\hat{\mu }}_{s},s=1,2\ldots ,S\right\}$. In the case of a single-injection, a generic time-course ($\bar{z}$) in $D_{S}$ is modelled ($\hat{\mu }$) by one of: (a) a scaled (venous) injection-site signal (*C*_*IV*_), (b) a non-parametric residue function (R), or (c), a scaled non-parametric distribution function (F) representing the total number of tracer atoms no longer in blood-tissue exchange as a function of time. This last case is to accommodate data in which the bladder is in the field of view. Thus

(1) $${\hat{\mu }}_{l}=\hat{A}\cdot C_{IV}\left(t_{l}-\hat{\Delta }\right)\;;\;{\hat{\mu }}_{l}=\hat{R}⋆C_{p}\left[t_{l}-\hat{\Delta }\right]\;;\;{\hat{\mu }}_{l}=\hat{F}⋆C_{p}\left[t_{l}-\hat{\Delta }\right]$$

for *l* = 1, 2, …, *N*_*T*_. Here *C*_*p*_ is the time-course of the tracer in the arterial blood (AIF). $\hat{\Delta }$ is an optimized delay factor. $\hat{R}$ and $\hat{F}$ are non-parametrically specified as piece-wise linear functions and estimated using constrained weighted least squares with weights $1/{\bar{\sigma }}_{l}^{2}$. It is important to appreciate that, apart from Δ, all unknowns in (1) are linear, so the computation of the optimal fit, for any fixed Δ, is evaluated by quadratic programming (QP). A grid search is used to determine the optimal delay. Choices other than the residue case are only selected if they significantly improve on the residue fit. Modelled elements are supplemented to include time-courses corresponding to the AIF and a further time-course corresponding to the integral of the AIF - the AIF exactly models a spike residue; the integral of the AIF corresponds to a constant residue. In deference to (Patlak and Blasberg; 1985), the latter component is referred to as the Patlak-element. With multiple injections an elaboration of (1) is used - details of this are indicated below.

**Step 2** of the basis selection procedure takes the full collection of modelled time-course patterns and applies a cross-validation guided backwards elimination procedure to identify a subset of these with the property that positive linear combinations of these patterns adequately fit the reduced dataset $D_{S}$. This leads to a final set of basis elements given by a *N*_*T*_ *× K* matrix $X=\left\{{\hat{\mu }}_{1},\ldots ,{\hat{\mu }}_{K}\right\}$. These basis elements ($\hat{\mu }$) are referred to as *sub-TACs* and *K* is their total number. Given the manner in which the segmentation process is implemented, voxel-level time-course data (*z*_*i*_) can be expected to be well-approximated by a positive linear combination of sub-TACs. NPRM fits voxel-level data by the linear model and approximates the voxel-level residue using the components that correspond to a residue process (1)

(2) $$z_{il}\approx \overset{K}{\underset{k=1}{\sum }}{\hat{\alpha }}_{ik}{\hat{\mu }}_{lk}=X{\hat{\alpha }}_{i}\;\to \;\hat{R}\left(t,x_{i}\right)=\underset{k\in K_{r}}{\sum }{\hat{\alpha }}_{ik}{\hat{R}}_{k}(t)$$

where $\hat{R}\left(t\cdot ,x_{i}\right)$ is the residue for the *i*’th voxel. Here *K*_*r*_ is the indices of the subset of sub-TACs that are specified by a residue in (1). QP is used to evaluate ${\hat{\alpha }}_{i}$.

#### 2.1.1. Residue Decomposition and Mapping of Kinetics:

Suppose a generic residue function *R* can be measured over a time interval [0, *T*_*E*_] - the PET scanning time-frame. Let *T*_*B*_ for 0 < *T*_*B*_ < *T*_*E*_ represent a realistic upper-bound for the large-vessel travel-time of tracer atoms - a *T*_*B*_ value of 15 seconds seems physiologically reasonable, for most PET tracers, including FDG and H_2_O. Following (O’Sullivan et al.; 2014), the residue can be written as a sum of vascular, in-distribution and extracted elements

(3) $$R(t)=R_{B}(t)+R_{D}(t)+R_{E}(t)$$

where *R*_*E*_(*t*) = *R*(*T*_*E*_) for *t* ∈ [0, *T*_*E*_]; *R*_*B*_(*t*) = *R*(*t*)*−R*(*T*_*B*_) for *t* ∈ [0, *T*_*B*_] and *R*_*B*_(*t*) = 0 for *t* > *T*_*B*_; *R*_*D*_(*t*) = *R*(*t*) − *R*_*B*_(*t*) − *R*_*E*_(*t*). Four useful summaries are

(4) $$V_{B}=\int_{0}^{T_{B}}R_{B}(t)dt\;;\;V_{D}=\int_{0}^{T_{E}}R_{D}(t)dt\;;\;K_{D}=R_{D}(0)\;;\;K_{i}=R_{E}(0)$$

These assess, large vessel and small vessel distribution volumes (*V*_*B*_, *V*_*D*_), non-large vessel flow (*K*_*D*_) and retention or apparent flux (*K*_*i*_) at the end of scanning period. Note, that all of these quantities are linear functions of the residue. Tracer extraction is measured as *K*_*i*_/*K*_1_ where *K*_1_ = *K*_*D*_ + *K*_*i*_ represents the overall flow. By the central volume theorem (Meier and Zierler; 1954), mean transit time (MTT) of non-extracted tracer is measured by a ratio of volume to flow. The measure may be adjusted to account for an overall delay (Δ) in the arrival of tracer atoms to the tissue region. This gives MTT = *V*_*D*_/*K*_*D*_ + Δ.

In the NPRM procedure voxel-level kinetic parameters are based on the estimated residue in (2). This leads to mapped values for (*V*_*B*_, *V*_*D*_, *K*_*D*_, *K*_*i*_, *K*_*i*_/*K*_1_). For MTT, we use $\frac{V_{D}}{K_{D}}+\bar{\Delta }$ where $\bar{\Delta }$ is the flow-weighted average of the delays (${\hat{\Delta }}_{k}$) associated with residue components at the voxel being mapped, *i.e*. at voxel *i*, $\bar{\Delta }=\frac{\sum_{k\in K_{r}}{\hat{\alpha }}_{ik}K_{Dk}{\hat{\Delta }}_{k}}{\sum_{k\in K_{r}}{\hat{\alpha }}_{ik}K_{Dk}}$ with *K*_*Dk*_ the flow for ${\hat{R}}_{k}$. Note (*V*_*B*_, *V*_*D*_, *K*_*D*_, *K*_*i*_) are linear and (MTT, *K*_*i*_/*K*_1_) are (smooth) non-linear functions of *α*-coefficients in (2).

#### 2.1.2. Why is the NPRM Approach Reasonable?

The NPRM approach has three important features: (i) In diverse tissue environments the formal theoretical support for compartmental models may be limited (even from an in-vitro stand-point), especially with more novel PET tracers. In this context a non-parametric approach has improved flexibility to adapt to the true (unknown) kinetics. (ii) NPRM can accommodate significant variations in the arrival of tracer to different parts of the field of view. This is accomplished by the inclusion of delays in the modelling of sub-TACs. (iii) NPRM gives an ability to adjust for potential artifacts associated with the bladder or the injection site within the scanner field of view. This is not important in the data presented in this paper but has relevance in abdominal scanning and in cases where there may be enhanced temporal scanning resolution.

An alternative non-parametric approach, based on a truncated singular value decomposition (SVD), has been used to map perfusion parameters with MR and CT data (Østergaard et al.; 1996; Konstas et al.; 2009). Unlike the method used here, the SVD approach does not constrain the target residue to be either positive or monotone decreasing. Also, it does not have a mechanism to address heterogeneity voxel-level data or indeed to accommodate artefacts associated with the retention of tracer in the bladder or signals associated with a venous injection site in the field of view. Further examination would be helpful in getting a detailed clarification of the performance of the truncated SVD approach relative to the NPRM technique. NPRM mapping would also seem to have potential for application to perfusion imaging with MR and CT. In addition, the approach may also have a role in quantitative analysis of digital subtraction angiography imaging (DSA) data - see (Liang et al.; 2019)

A small 1-D PET-FDG numerical simulation study comparing NPRM with classic voxel-by-voxel compartmental analysis (Kety and Schmidt; 1948; Phelps et al.; 1979) is implemented. Details of the scanning model and source distribution are as specified in section 4. The source distribution is specified as a linear combination of specified sub-TACs (*μ*_*k*_) - like (2). In one case (I), source sub-TACs correspond to non-parametric residues derived from regional analysis of breast cancer imaging data; in the second case (II) source sub-TACs are replaced by the best fitting one-compartment (1C) model (Kety and Schmidt; 1948) to the real data. The table reports the relative root mean square error (RMSE) performance of mapping the six summary kinetic parameters described above, when voxel-level residues are estimated using either the NPRM technique or by application of a 2-compartment (2C) model (Phelps et al.; 1979) that includes adjustment for the fractional blood volume and tracer delay (O’Sullivan et al.; 2009). The model function (*η*) is:

(5) $$\eta (t|\beta )=f_{b}C_{p}(t-\Delta )+\int_{0}^{t}\left(A_{1}e^{-\lambda_{1}(t-s)}+A_{2}e^{-\lambda_{2}(t-s)}\right)C_{p}(s-\Delta )ds$$

where *C*_*p*_ is the AIF and *f*_*b*_ accounts for the contribution from the vasculature. The unknown parameters are *β*= (Δ, *f*_*b*_, *A*_1_, *A*_2_, *λ*_1_, *λ*_2_) – Δ is unconstrained but the other components of *β* are constrained to be positive.

For computational reasons, associated with the 2C model fitting, RMSE values are based on *N*_*R*_ = 50 replicate simulations at each dose. Relative RMSE values, ${\hat{e}}_{ipd}$ in (6), are consistent across *N*_*d*_ = 5 dose levels (*d*) and *N* voxels (*i*). Average values and standard errors are summarized for each of the six parameters (*p*) - (*V*_*B*_, *V*_*D*_, *K*_*D*_, *K*_*i*_, *MT T*, *K*_*i*_/*K*_1_).

(6) $${\hat{e}}_{p}=\frac{1}{NN_{d}}\underset{i,d}{\sum }{\hat{e}}_{ipd}\;;\;p=1,2\ldots ,6\text{ where }{\hat{e}}_{ipd}=RMSE_{ipd}^{2C}/RMSE_{ipd}^{\text{NPRM}}$$

Table 1 presents the comparisons between the NPRM and 2C methods when the source sub-TACs are generated by a compartmental form (case II) and also when the sub-TACs follow a non-parametric form (case I). Results show substantially enhanced performance of NPRM when the source is not compartmental and a more similar performance when the source is compartmental. Remarkably however, perhaps due to spatial mixing associated with data reconstruction, the 2C method does not perform as well for the *V*_*B*_ and *V*_*D*_ parameters. Indeed NPRM is seen to be strongly competitive with the 2C mapping scheme when the underlying source is specified in terms of a compartment model structure. At its worst, in the case of *K*_*i*_/*K*_1_, the relative RMSE efficiency of NPRM mapping is 84%. This result is quite typical.

#### 2.1.3. Local Error Characteristics of NPRM Mapping:

Although the voxel-level model in (2) used by NPRM has a linear form, the sub-TACs involved, $X=\left\{{\hat{\mu }}_{1},\ldots ,{\hat{\mu }}_{K}\right\}$, are derived by a complex data-adaptive process. Hence the overall error in voxel-level kinetics is a function of the uncertainties in the estimation of *α*-coefficients *and* the uncertainties in the construction of sub-TACs. Intuitively, because the sub-TACs are constructed by modelling average time-course data for segments, one might expect that the uncertainties associated with the sub-TACs have a minor impact on the error on NPRM voxel-level kinetics. In order to justify this intuition, we report on another simulation. Here we used a standard 2D PET-FDG simulation in which voxel-level kinetics were assessed using both known and unknown data-estimated sub-TACs. The 2-D context is used as the segmentation process with 2-D data gives rise to more realistic subsets of data defining each sub-TAC. The scanning model and source structure are as described in section 4. The statistical relation between the RMSE (averaged across voxels) using known and unknown sub-TACs are shown in Table 2. This study only considered a single (realistic) dose and was restricted to a modest number of replications (*N*_*R*_ = 50) - standard errors of simulation estimated quantities of interest do not suggest we need any more. The results show that kinetic mapping errors are substantially the same regardless of whether or not sub-TACs are known. Thus when analyzing voxel-level NPRM kinetic mapping errors, it seems sufficient to focus on error associated with determination of voxel-level *α*-coefficients conditional on known model sub-TACs. We will adopt this approach in our analysis of the impact of data combination.

We now focus on what controls NPRM kinetic mapping error. A simplistic linear model analysis (Seber; 2009) of the NPRM model (2) would suggest that the RMSE of the kinetic summaries should substantially scale with the level of uncertainty in the reconstructed data. Thus for a given voxel (*i*) the error in an estimate of kinetic variable, ${\hat{\theta }}_{ip}$, denoted ${\text{RMSE}}_{ip}^{\text{NPRM}}$ should have a strong relation to the corresponding root-mean-square deviation between the reconstructed time-course data (*z*_*il*_) and the true voxel-level time course $\left(\lambda_{il}\right)-RMSE_{i}^{z}=\sqrt{\frac{1}{N_{T}}\sum_{j=1}^{N_{T}}{\left[z_{il}-\lambda_{il}\right]}^{2}}\equiv {\hat{\sigma }}_{i}$. We examined this numerically using the simulation data used to produce Table 1. The statistical analysis, which incorporated data from a range of dose settings, finds

(7) $$\text{log}\left(RMSE_{ip}/\theta_{ip}\right)\approx \alpha_{p}+\beta_{p}\text{ log}\left({\hat{\sigma }}_{i}/\theta_{ip}\right)$$

well-describes the relation between voxel-level errors in kinetics and the local reconstruction accuracy of the input data (${\hat{\sigma }}_{i}$). Model *R*^2^-values and *β*-coefficients are given in Table 3. Note that (7) suggests formal mean square error convergence of the NPRM procedure - as the reconstruction error diminishes so too does the error in determination of kinetics. The rate of convergence, measured by the *β*_*p*_ coefficient, is seen to be close to 1 and suggests that standard parametric inference asymptotics apply to this situation (Van der Vaart; 2000). A similarly strong relation is found between the modelling error associated with the linear model (2), ${\text{RMSE}}_{i}^{\hat{\lambda }}=\sqrt{\frac{1}{N_{T}}\sum_{j=1}^{N_{T}}{\left[{\hat{\lambda }}_{il}-\lambda_{il}\right]}^{2}}\equiv {\hat{\sigma }}_{i}^{\lambda }$, and the RMSE of the kinetic summaries, *i.e*.

(8) $$\text{log}\left(RMSE_{ip}/\theta_{ip}\right)\approx \alpha_{p}^{\prime }+\beta_{p}^{\prime }\text{ log}\left({\hat{\sigma }}_{i}^{\lambda }/\theta_{ip}\right)$$

In simulations this is important as it allows us to use modelling error as a surrogate metric for the analysis of errors in individual kinetic variables.

### 2.2. Multiple Injections and their Combined or Separated Analysis by NPRM

With multiple injections the model in (1) is elaborated to account for the effect of individual injections and the separate delay factors that may be associated with measurement of the tracer AIFs. This gives

(9) $${\hat{\mu }}_{k}=\overset{J}{\underset{j=1}{\sum }}{\hat{A}}_{j}\cdot C_{IV}^{\left(j\right)}\left(t_{k}-{\hat{\Delta }}_{j}\right)\;;\;{\hat{\mu }}_{k}=\overset{J}{\underset{j=1}{\sum }}{\hat{R}}_{j}⋆C_{p}^{\left(j\right)}\left[t_{k}-{\hat{\Delta }}_{j}\right]\;;\;{\hat{\mu }}_{k}=\overset{J}{\underset{j=1}{\sum }}{\hat{F}}_{j}⋆C_{p}^{\left(j\right)}\left[t_{k}-{\hat{\Delta }}_{j}\right]$$

Here the index *j* relates to individual injections. As with (1), the residue and outflow functions can either be modelled using suitable parametric forms (Kadrmas and Hoffman; 2013; O’Sullivan; 1993; Spence et al.; 1998) or with non-parametric formulations as used by (Lan et al.; 2019). Note the non-parametric approach leads to an optimization problem, in which only the delay factors Δ_*j*_ enter in a non-linear fashion. This simplifies computation. Our interest is in the case where it is possible to consider the separate analysis of kinetics of individual injections. In this setting, because there is negligible temporal overlap (spillover) between the separate injections, modelling of sub-TACs in the NPRM voxel model can be accomplished by separate application of the simple form in (1). Regardless of whether there is overlap or not, voxel-level kinetic parameters, say {*θ*_*p*_, *p* = 1,2, … *P*}, are expressed as simple functions of the *α*-coefficients in (2). Thus ${\hat{\theta }}_{ip}=g_{p}\left({\hat{\alpha }}_{i}\right)$ where *g*_*p*_ is a (smooth) function defined using the estimated residue functions and delay factors for separate injections.

If data are combined, coefficients are obtained based on the full time-course for the measured data and the sub-TACs. Separate analysis recovers kinetics based on the shorter sub-TAC time-course corresponding to individual injections. Now suppose we are interested in a kinetic summary variable corresponding to the *j*’th tracer injection - say *θ* = *g*(*α*). The separate analysis estimate, ${\hat{\theta }}^{j}=g\left({\hat{\alpha }}^{j}\right)$, is based on fitting (2) using only the *j*-injection time-course; the combined estimate, ${\hat{\theta }}^{C}=g\left({\hat{\alpha }}^{C}\right)$, is based on fitting the full time-course data. Thus at the *i*’th voxel we would map the kinetic parameter associated with *g* using one of

(10) $${\hat{\theta }}_{i}^{j}=g\left({\hat{\alpha }}_{i}^{j}\right)\text{ or }{\hat{\theta }}_{i}^{C}=g\left({\hat{\alpha }}_{i}^{C}\right)$$

Intuitively, we expect that the combined data estimator will be preferred but it is helpful to have a quantitative appreciation of the factors that might be important. The results reported in the paper address this issue via numerical simulation. Before that, we present a simplified theoretical analysis that may give some intuition.

### 2.3. An Approximate Theoretical Analysis of Combined versus Separated Analysis

Table 3 suggests that regular parametric statistical inference theory associated with Gaussian approximation applies to the NPRM model coefficient estimates and the derived kinetic variables. In light of this it seems reasonable to use Gaussian approximation to develop insight into the potential benefits of combined versus separate analysis for multi-injection studies. By Gaussian approximation we get the following.

#### Result 2.1.

*If the conditions for Gaussian approximation hold*, *the combined analysis estimator always has lower variance than the separate injection estimator. For a given kinetic parameter (θ)*, *the ratio of the MSE of the combined analysis estimate*, *based on J injections*, *to the MSE of the separate analysis estimate*, *using only study j*, *is given by the formula*

(11) $$\frac{\text{MSE}\left({\hat{\theta }}^{C}\right)}{\text{MSE}\left({\hat{\theta }}^{j}\right)}=\frac{\sigma_{j}^{-2}}{\sigma_{1}^{-2}+\sigma_{2}^{-2}+\ldots +\sigma_{J}^{-2}}$$

where $\sigma_{j}^{2}$ is the variance of the kinetic parameter based on data from injection j.

The Appendix provides a theoretical justification for this result. In replicate studies, individual scan variances (*σ*_*j*_) are identical so the MSE ratio is 1*/J*, *i.e*. the percent improvement in MSE with the combined analysis is proportional to (*J* − 1). While the combined analysis will always produce better estimates, the amount of improvement will depend on the reliability of the other scans involved. (8) indicates that the reliability of kinetics for individual scans kinetics is a function of the associated source model error. We would anticipate that the study dose and relative sharpness of the AIF, which impact voxel-level model error, would play a role. Numerical studies will explore this in some detail.

## 3. Illustration with a Flow-Metabolism Study in a Breast Cancer Patient

The data come from a study conducted on a breast cancer patient prior to surgical resection and prior to scheduled neoadjuvant chemotherapy. PET scanning involved dynamic imaging with ^15^O-H_2_O and ^18^F-FDG in the same session. ^15^O-H_2_O is a tracer used to measure blood flow and perfusion - the gold-standard technique for *in-vivo* measurement of such information. ^18^F-FDG is the most widely used clinical PET tracer. FDG measures information about tissue glucose utilization. It is a key marker used in the diagnosis and clinical management of several cancers. Details of PET dynamic imaging protocols for H_2_O-FDG scanning in breast cancer and reports on the prognostic utility of the derived information are given in the earlier reports - see (Mankoff et al.; 2002, 2003; Dunnwald et al.; 2008, 2011). For the data we present here, 1302 MBq of ^15^OH_2_O in a 1–4 mL volume was injected as a bolus and 318 MBq of ^18^F-FDG in 7–10 mL volume was injected over 2 minutes with a constant infusion pump. Data were acquired on a GE-Advance scanner using a plane-by-plane FBP reconstruction algorithm with corrections for attenuation, scatter, deadtime and random events. The 4-D PET data set consists of an imaging volume with *N* = 128 × 128 × 35 voxels and *T* = 82 (57 for H_2_O and 25 for FDG) time frames of acquisition. Dynamic ^15^O-H_2_O images were collected for 8.75 min according to the following acquisition sequence (number of time frames and their durations): 1 × 1 min (pre-injection), 15 × 2 s, 15 × 5 s, 12 × 10 s, 8 × 15 s and 6 × 20 s. The FDG injection and dynamic imaging followed H_2_O. The FDG acquisitions were conducted over 61 minutes according to the sequence: 1×1 min (pre-injection), 4 × 20 s, 4 × 40 s, 4 × 1 min, 4 × 3 min and 8 × 5 min.

The data were analyzed using the NPRM technique described in section 2. Arterial input functions used were recovered from the left-ventricle (LV) of the heart using the technique reported in (O’Sullivan et al.; 2017). Kinetics were mapped using both separate and combined analysis of the H_2_O and FDG data.

**Figure 3:**
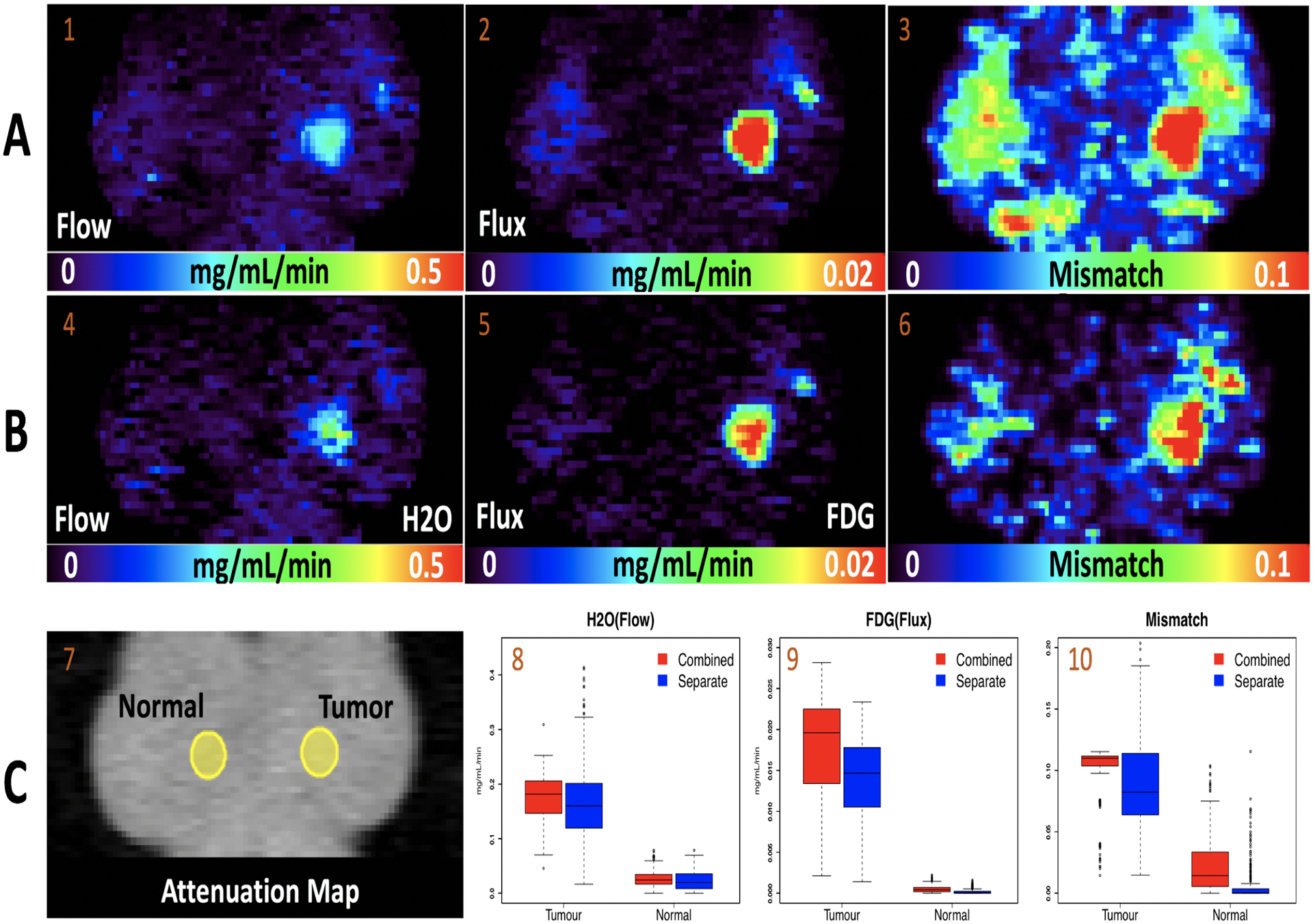
Estimates of flow, flux and mismatch in a H_2_O-FDG breast cancer study from combined and separate analysis. **Row A:** Combined Analysis; **Row B:** Separate Analysis; **Row C:** ROI Analysis.

This analysis provides voxel-level mapping of H_2_O blood flow ($K_{1}^{O}$, mg/mL/min), FDG metabolic rate or flux ($K_{i}^{F}$, mg/mL/min) and metabolism-flow mismatch ratio ($K_{i}^{F}/K_{1}^{O}$) for separate or combined analysis approaches. Results are presented in Figure 3. Coronal planes of metabolic parameters demonstrate increased flow and metabolism in the tumour. The flow-metabolism mismatch is also seen to be highly elevated in the tumor region - an elevated mismatch has been shown to be associated with poor response to chemo-therapy and early cancer relapse (Mankoff et al.; 2002). In qualitative terms the flow and mismatch maps recovered by separate analysis appear more noisy, the flux maps are not much different. To explore these differences more carefully, two regions-of-interests (ROIs) - normal breast (335 voxels) and tumour (322 voxels) are extracted, as shown in Figure 3. The distribution of metabolic parameters in these two ROIs is presented in boxplots in Figure 3.

We can see the flow distributions in tumour and normal breast are more variable for the separate analysis, the standard deviations (SD) of flow from combined and separate analysis in 2 ROIs are 0.04 and 0.07 (tumour), 0.014 and 0.018 (normal breast) respectively. The additional variability is on the order of 75% and 28.6% in tumour and normal breast. For flux, the standard deviations with the two approaches are 0.006 and 0.005 (tumour), 0.0004 and 0.0003 (normal breast). These differences are small. The variability of mismatch from separate analysis is much bigger than the combined analysis in the tumour region - the situation is somewhat reversed in the normal region but this could also be due to an underestimation of the normal tissue flow in the separate analysis. In light of these results, we are motivated to develop a better understanding of the relative statistical behaviour of combined and separate analysis of this and similarly structured multiple injection studies.

## 4. Numerical Experiments

**Figure 4:**
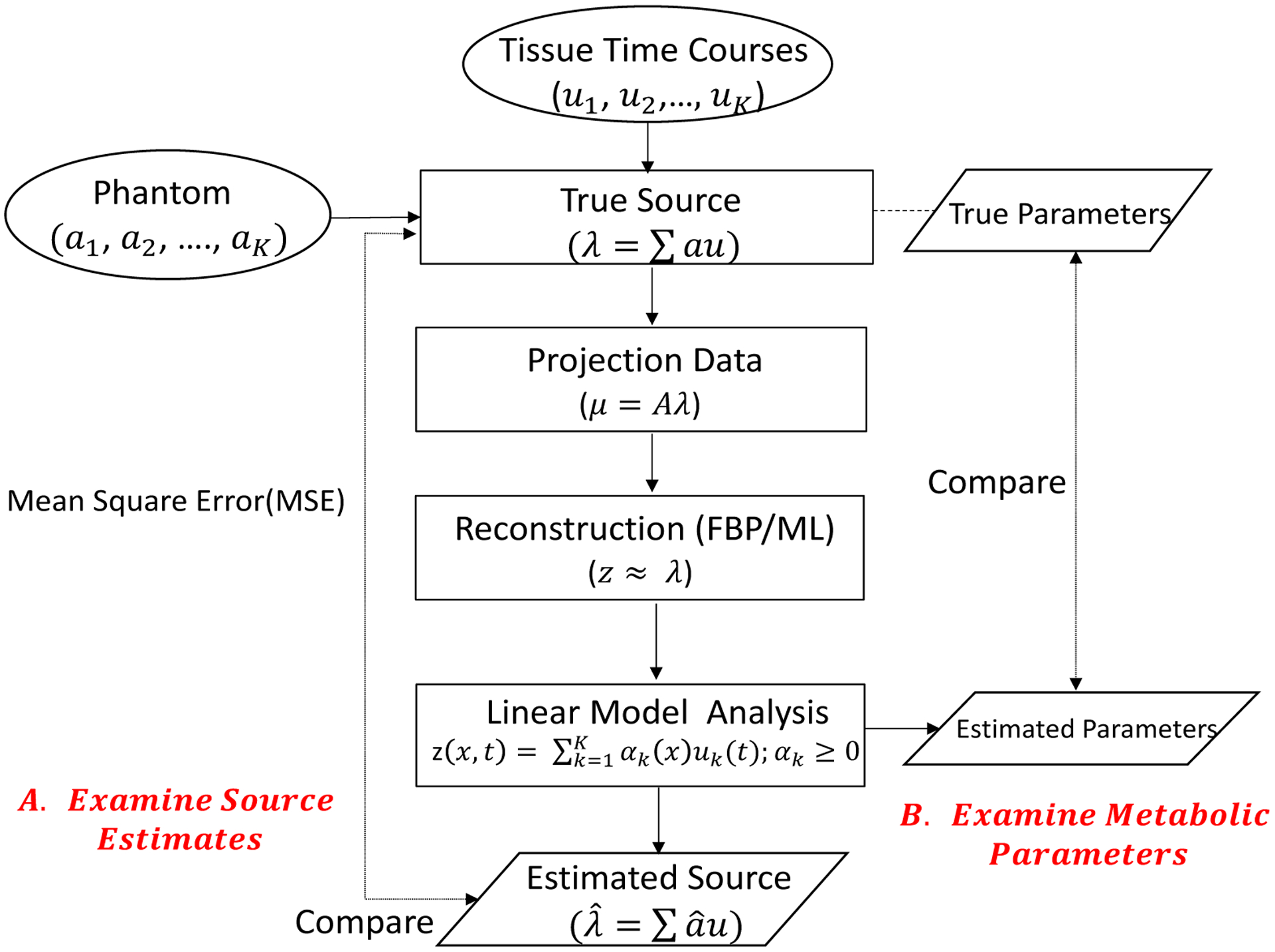
Diagram of Simulation Process

We conducted two sets of experiments, one related to the H_2_O-FDG study in section 3 and a second one relating to repeat H_2_O activation studies. A schematic for the structure of the simulation process is provided in Figure 4. Below we provide details of the setup of the 1-D and 2-D simulation models used, the specification for the source distributions in the two examples and, finally, the procedure used to compare the performance of the combined and separate analysis techniques. FBP and ML reconstructions are both considered in 1D simulation studies, but for computational efficiency we restrict the H_2_O-FDG setup to only FBP reconstruction in the 2-D case.

### 4.1. Scanning Models

In the 2-D case a standard PET scanning model involving Poisson sampling of a discretized (attenuated) parallel-beam Radon transform of the source is used (Kak et al.; 2002; Natterer; 2001). The imaging domain is the unit square, discretized to an array of dimension 128 × 128, and the projection domain is the region $\left[-\sqrt{2},\sqrt{2}]\times [0,\pi \right]$, discretized to a 183 × 181 sinogram array of distances and angles. For computational reasons numerical experiments in 2-D only consider direct FBP reconstruction. In 1-D a less familiar Poisson deconvolution model is employed. The model is used because the computational efficiencies enable us to carry out a sufficient number of replicate studies using both direct and iteratively reconstructed data. Full details of the model are given in (O’Sullivan and Roy Choudhury; 2001). The observed data over a given time-frame is a realization from a projected and scaled source. The projection process is given by the product of a fixed attenuation profile (*a* > 0) and the discrete convolution between the scaled source and a kernel *κ*^*β*^ (*β* > 0). The kernel acts to smooth the source - its discrete Fourier transform is given by

$$\kappa_{\nu }^{\beta }=|\nu {|}^{-\beta }\text{ for }\nu =\pm 1,\pm 2\ldots N/2.$$

If *y* is a realization from the projected and scaled source then the direct reconstruction of *y* is simply obtained

$$z^{*}=\frac{1}{\tau }F^{-1}\left\{|\nu {|}^{\beta }{\hat{\left(y/a\right)}}_{\nu }\right\}$$

where ($\hat{y/a}$) is the discrete Fourier transform of the attenuation corrected count data, $F^{-1}$ represents the inverse transform and *τ* is the expected total counts (dose) used for scaling. Smoothing is achieved by convolving the raw reconstruction z* with a discrete Gaussian kernel. The smoothed reconstruction is an analogue of an FBP reconstruction used in PET. ML reconstructions are readily obtained in this setting. Standard iteratively re-weighted least squares (IRLS) (with positivity constraints) apply. The convolution structure allows such iterates to be easily evaluated. Similar to FBP, the raw ML estimator can be smoothed to obtain an analogue of the expectation–maximization (EM) reconstruction methods used in PET. The simple convolution model captures the essential estimation complexity of PET. This is because the reconstruction filter acts like a fractional derivative. By choice of *β*, it is possible to adapt the model so that the behaviour of the reconstruction MSE as a function of count rate, is matched to 2-D PET. Experimentation by Gu (Gu; 2021) with the breast FDG data in section 3 found that a value of *β* = 1:35 in the 1-D scanning model worked well. This is the setting we use in the studies reported here.

### 4.2. Source Patterns

The true (target) source is expressed as a linear model with *K* components.

(12) $$\lambda (x,t)=\alpha_{1}(x)\mu_{1}(t)+\alpha_{2}(x)\mu_{2}(t)+\ldots +\alpha_{K}(x)\mu_{K}(t)$$

**Figure 5:**
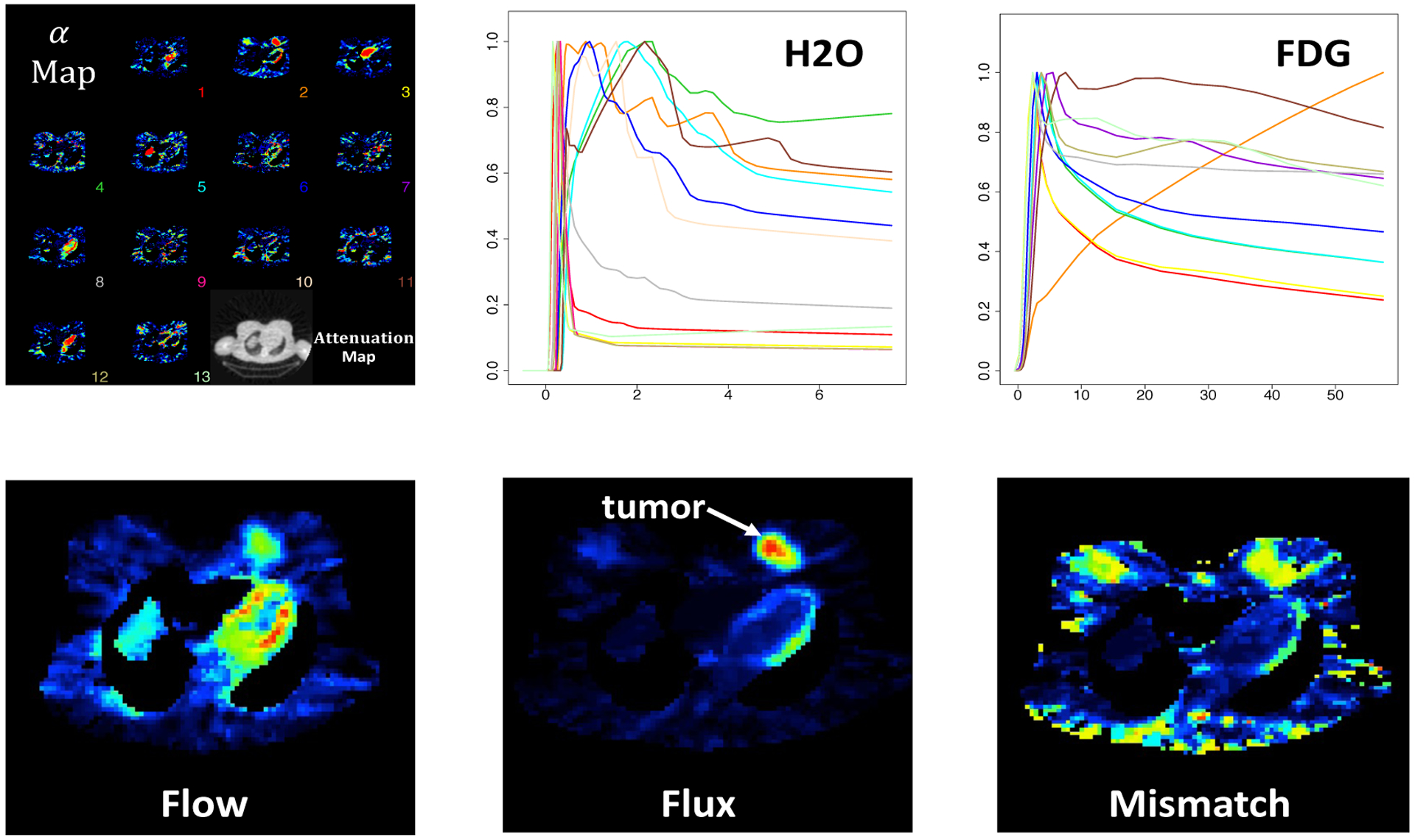
Source distribution, $\lambda_{xt}=\sum_{k=1}\alpha_{k}(x)\mu_{k}(t)$ for 2D simulations. **Row 1:** *α* map and normalized time courses (*μ*) for H_2_O and FDG. **Row 2:** True parametric images for flow, flux and mismatch.

In the 2-D case we only consider the H_2_O-FDG studies. The configuration is matched to the real data presented in section 3 - see Figure 5. Spatial patterns correspond to a central slice through the tumor region; the temporal patterns including the AIF come from the full data set.

**Figure 6:**
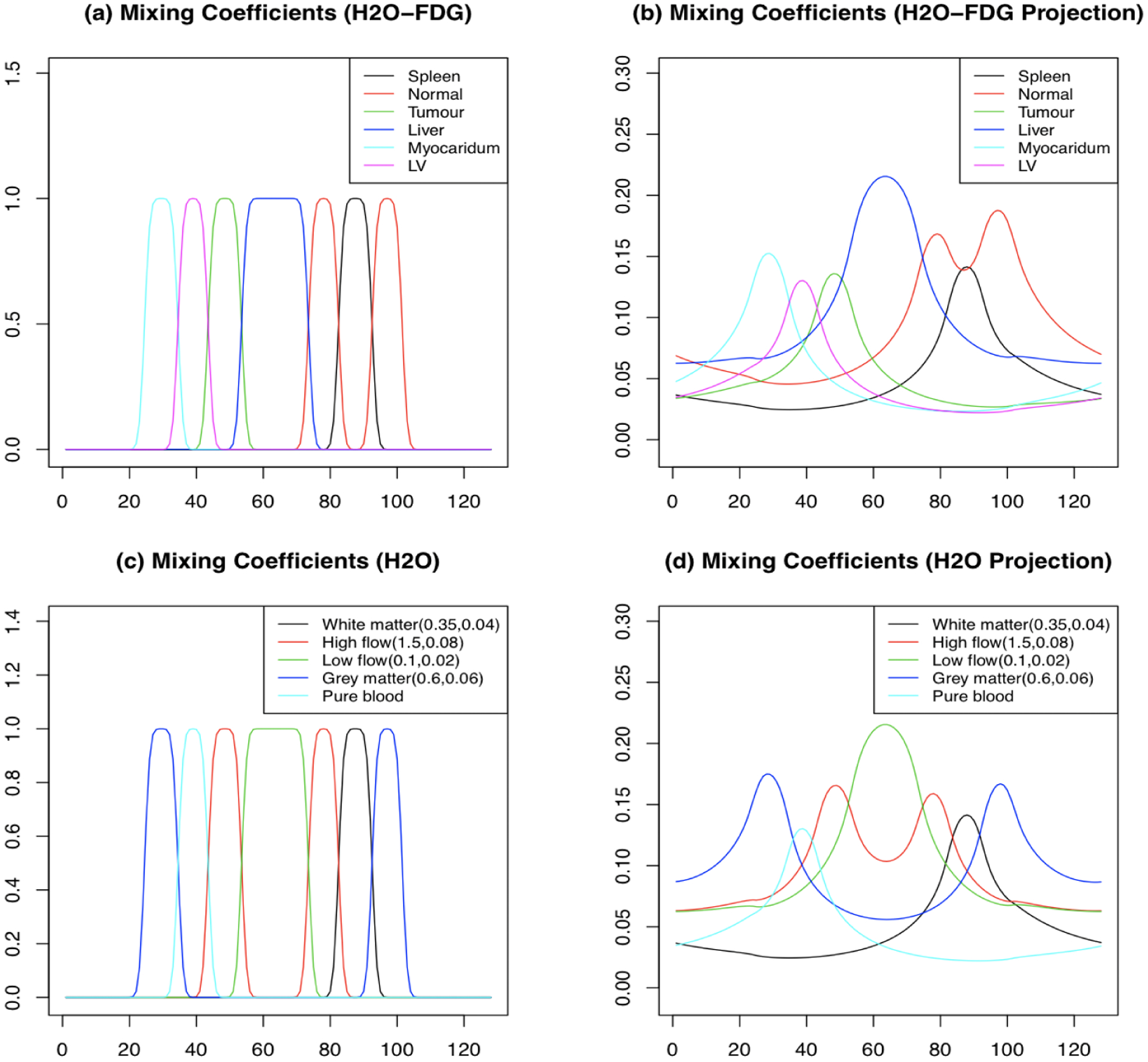
1-D phantom (mixing coefficients - *α*_*k*_’s) and their projections for H_2_O-FDG (a,b) and repeat H_2_O studies (c,d). x-axis is location (1–128) and y-axis is coefficient. Different colors represent different ROIs. Values of the Kety model parameters (*K*_1_, *K*_1_/*k*_2_) used to create simulated time courses are given in the legends for (c) and (d).

**Figure 7:**
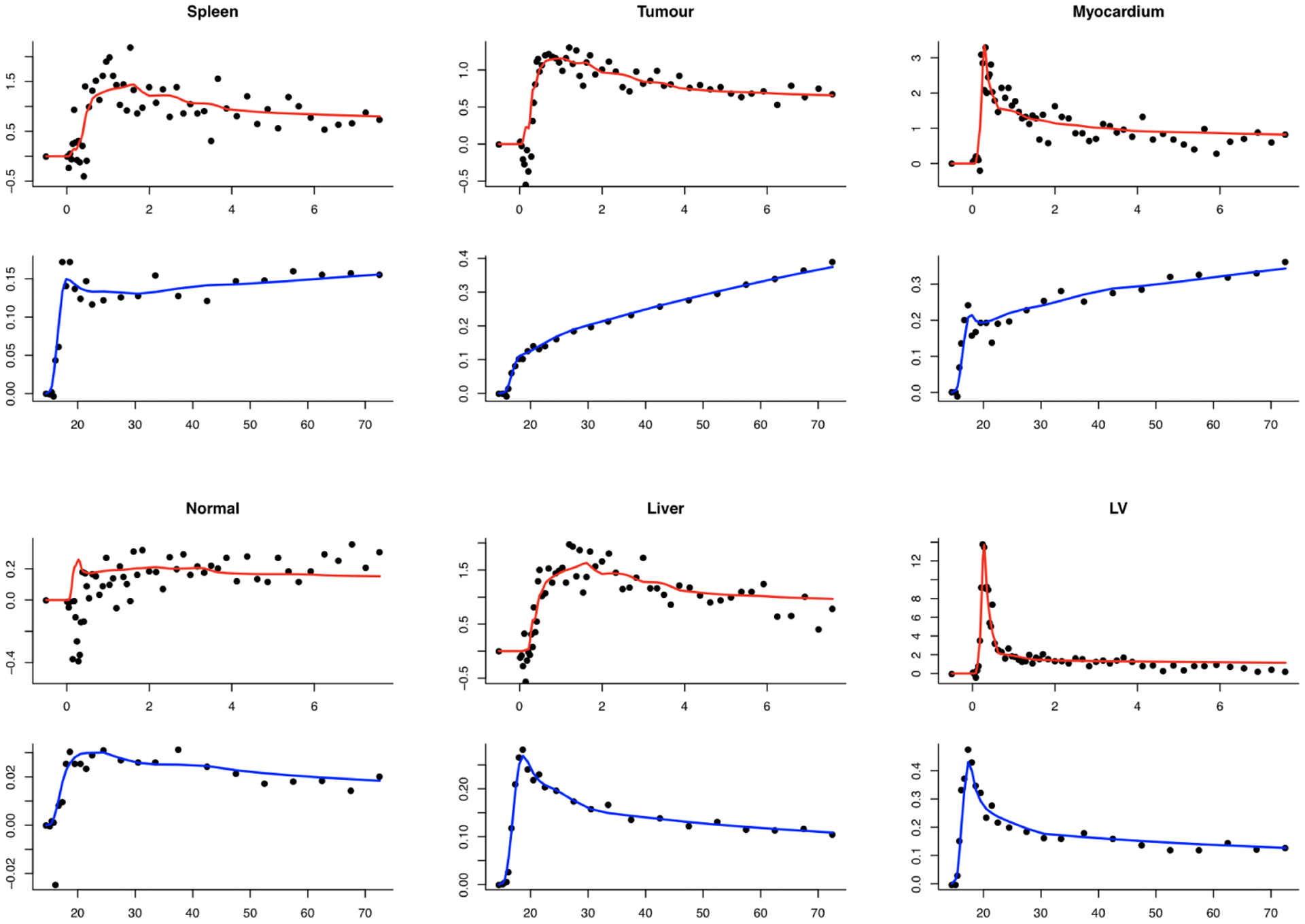
Fitted time courses and real data for six kinds of tissues - spleen, tumour, myocardium, normal breast, liver and left ventricle (LV). Red and blue lines are fitted time courses for the H_2_O and FDG studies, respectively. Black points are real data. x and y axis are time (minutes) after injection and activities (MBq/cc).

**Figure 8:**
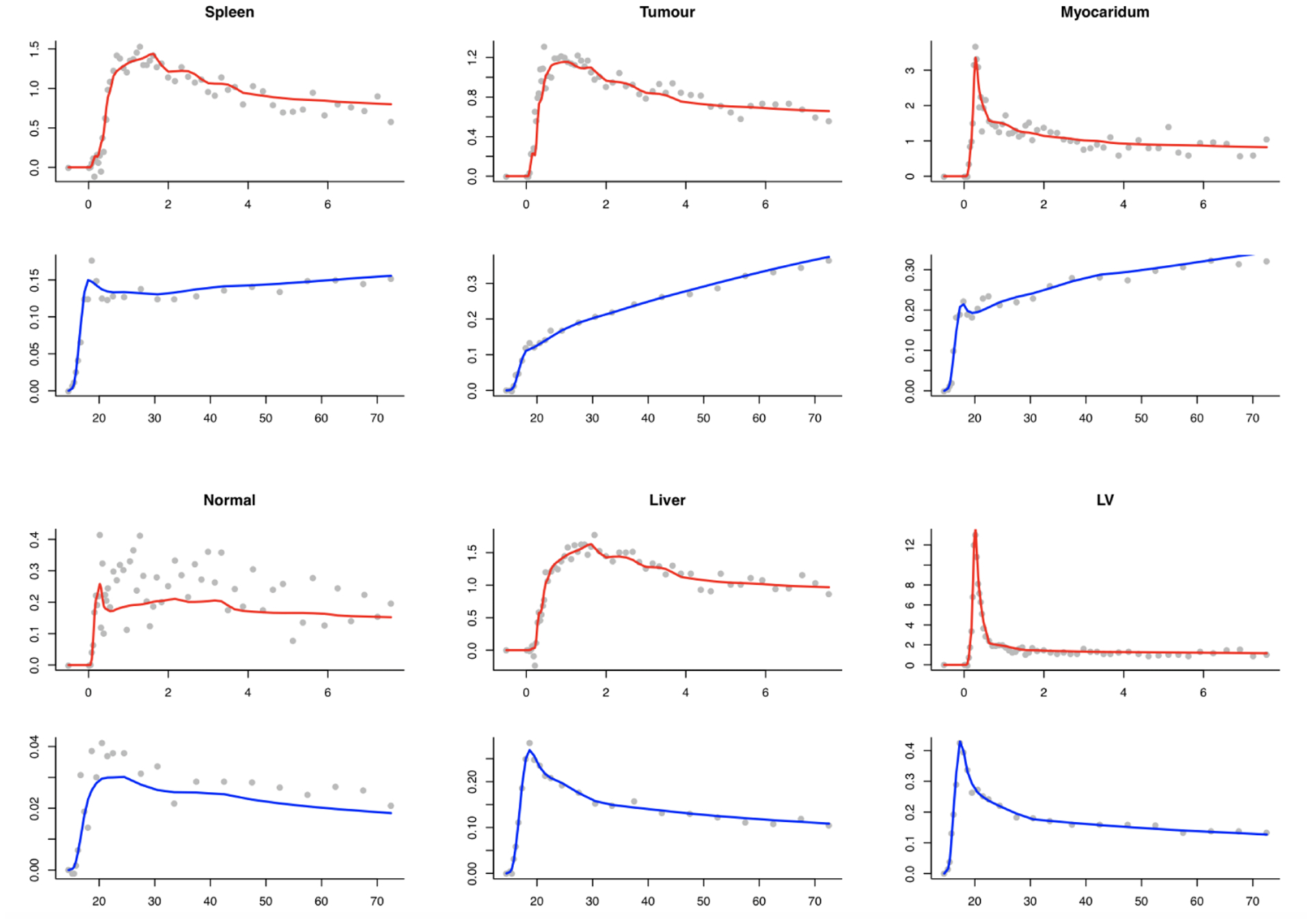
True time courses and simulated image data at middle dose and dose ratio for same six kinds of tissues. Red and blue lines are true time courses for H_2_O and FDG studies in simulation, respectively. Grey points are simulated data. x and y axis are time (minutes) after injection and activities (MBq/cc).

The source specification is more abstract in the 1-D case. Figure 6 shows the spatial pattern of the *α*-coefficients and how they are transformed into the measurement domain. This aspect is the same for H_2_O-FDG and the repeat H_2_O studies. The shape of the AIFs for H_2_O and FDG match those in the breast cancer data. The time-course patterns for the H_2_O-FDG are also adapted from the breast cancer analysis; in the repeat H_2_O studies, a 1-compartmental Kety-Schmidt (Kety and Schmidt; 1948) model is used to create patterns relating to regions in a normal brain - parameters are given in Figure 6. Figure 7 shows observed time course patterns for H_2_O and FDG from six ROIs in the breast data, together with the fit corresponding to the analysis in section 3. The time courses fitted by NPRM are used. The simulation uses a range of seven dose levels *τ*. The range is adapted to produce data with a voxel-level noise pattern matched to real data. For the H_2_O-FDG studies, the relative dose of the H_2_O and FDG injections are also varied. A range of seven dose-ratio settings from 16 : 1 to 64 : 1 are examined. The middle dose ratio corresponds to the data in section 3. Simulated data, at the middle dose and dose ratio for the H_2_O-FDG studies, are in Figure 8. The simulation noise level is a reasonable match to that seen in the real data. The repeat H_2_O study is calibrated in the same way, but not presented. In repeat H_2_O studies we consider situations in which there are 2, 4, 6 and 8 separate H_2_O injections. In 1-D studies with FBP and ML reconstructed data are reported. All studies, both for the H_2_O-FDG and repeat H_2_O, are replicated 500 times for each dose and in the H_2_O-FDG case for each dose-ratio setting.

### 4.3. Evaluation of Performance

Our studies use the model MSE characteristics of the estimated source, as a surrogate for understanding the MSE characteristics of estimates of the mapped kinetics. The motivation for this comes from Table 3 which showed a setting where MSE characteristics of mapped kinetics are proportional to the MSE reliability of the underlying source estimate. As indicated in Figure 1 the overall scan period, *T*, is made up of a set of *J* non-overlapping time intervals corresponding to the scanning periods for the separate tracer injections under consideration, *T* = *T*_1_∪*T*_2_ ⋯∪*T*_*J*_. If ${\hat{\alpha }}^{C}$ is the combined estimate coefficient, the corresponding estimated source is ${\hat{\lambda }}^{C}(x,t)=\sum_{k=1}^{K}{\hat{\alpha }}_{k}^{C}(x)\mu_{k}(t)$ and the squared error assessment is

(13) $$Se\left({\hat{\lambda }}^{C},\lambda \right)=\int_{\Omega_{x}}\underset{j}{\sum }\frac{1}{D_{j}{\bar{\lambda }}_{j}^{2}}\int_{T_{j}}{\left[{\hat{\lambda }}^{C}(x,t)-\lambda (x,t)\right]}^{2}\text{dtdx}$$

where *D*_*j*_ and ${\bar{\lambda }}_{j}$ are the durations and maximum intensities for the *j*′th scan. Integrals are evaluated by simple discretization. For the separate scan case, the estimate of the source for the *j*’th time-interval is based on the corresponding coefficients (${\hat{\alpha }}^{j}$) - *i.e*. ${\hat{\lambda }}^{S}(x,t)=\sum_{k=1}^{K}{\hat{\alpha }}_{k}^{j}(x)\mu_{k}(t)$ for *t* ∈ *T*_*j*_. In this way an overall assessment for the separate scan analysis is obtained as $Se\left({\hat{\lambda }}^{S},\lambda \right)$. With simulated data, we evaluate the error of combined and separated scan estimators for study replicates and by averaging an MSE assessment for the source error is obtained. This gives

(14) $$MSE^{C}=E(Se\left({\hat{\lambda }}^{C},\lambda \right)\text{ and }MSE^{S}=E(Se\left({\hat{\lambda }}^{S},\lambda \right)$$

Relative percent improvement is assessed by

(15) $$\%\text{Improvement}=\frac{MSE^{S}-MSE^{C}}{MSE^{C}}\times 100\%=\left(\frac{MSE^{S}}{MSE^{C}}-1\right)\times 100\%$$

## 5. Results

We begin by presenting some sample results for the H_2_O-FDG and repeat H_2_O studies. This is followed by detailed MSE comparisons, including some associated plots.

### 5.1. Sample Metabolic Parameters Estimates

**Figure 9:**
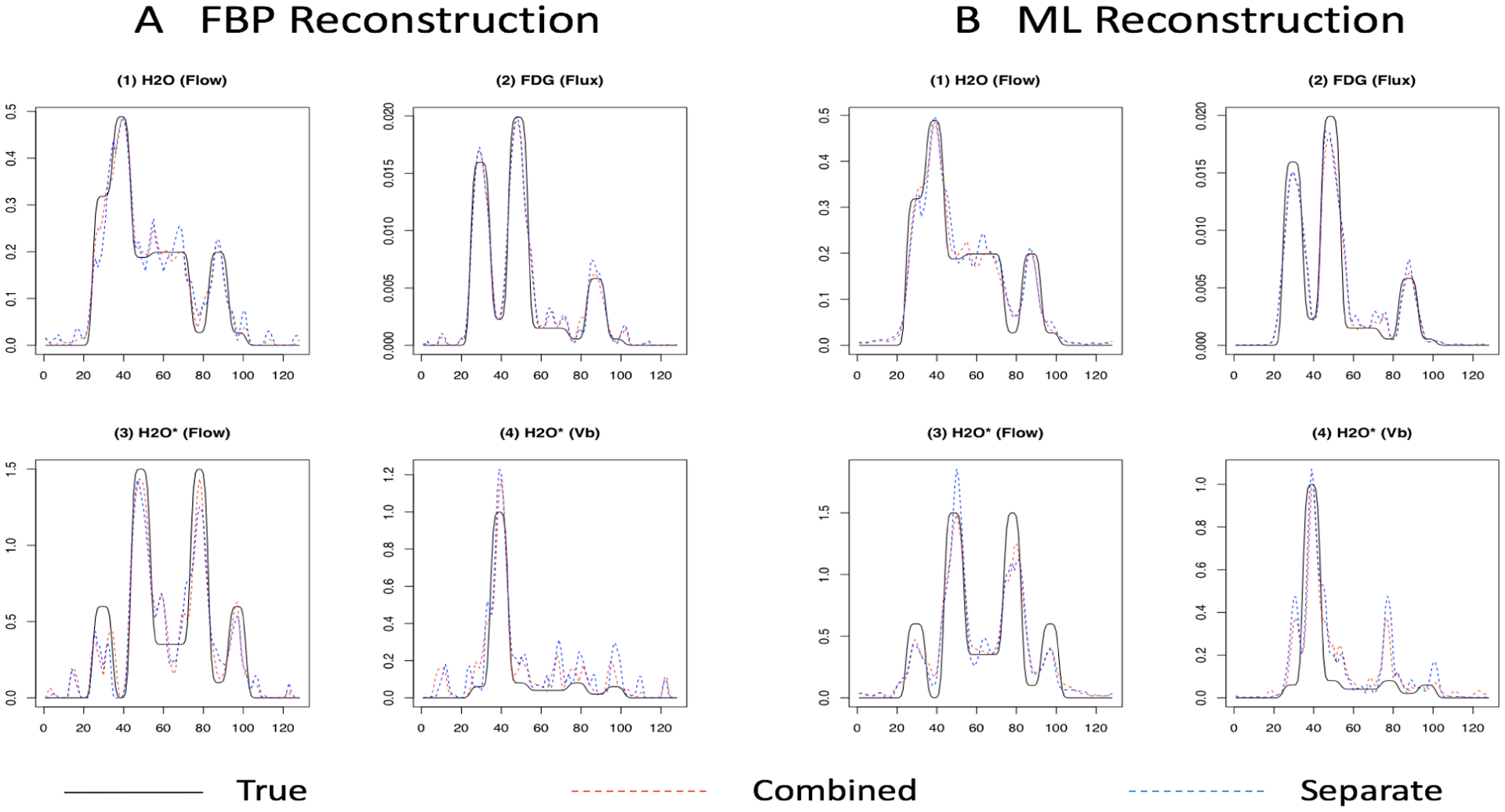
Sample metabolic estimates (at a middle dose setting) based on the FBP and ML reconstruction. x-axis gives location (1–128); y-axis is values of metabolic parameters in 1-D simulations. See Figure 6 for location of different structures. Plots (1) and (2) show flow and flux in H_2_O-FDG study; (3) and (4) show flow and blood volume (*V*_*B*_) in the repeat 2 H_2_O studies.

In the breast cancer study, H_2_O and FDG are used to measure blood flow and glucose metabolism, respectively. Metabolic parameter comparisons for a single replicate at the middle dose and dose-ratio are presented in Figure 9. True parameters are represented by black lines. Estimated parameters from combined and separate analysis are shown using dashed red and blue lines separately. We can see metabolic estimates from combined analysis are closer to the true parameters and show more advantages in H_2_O study. Metabolism estimated from these two approaches are both close to the true values in FDG study and difference can be negligible. The quantitation of H_2_O can get more benefits from the combined analysis and these results are also consistent with findings from the real dataset in section 3. Similarly, we also show one sample metabolic parameters - flow and blood volume (*V*_*B*_) at the middle dose in a repeat H_2_O study (H_2_O*) in Figure 9. The combined analysis is seen to achieve improvements in both situations.

### 5.2. MSE Performance Comparisons

#### 5.2.1. H_2_O-FDG Studies:

**Figure 10:**
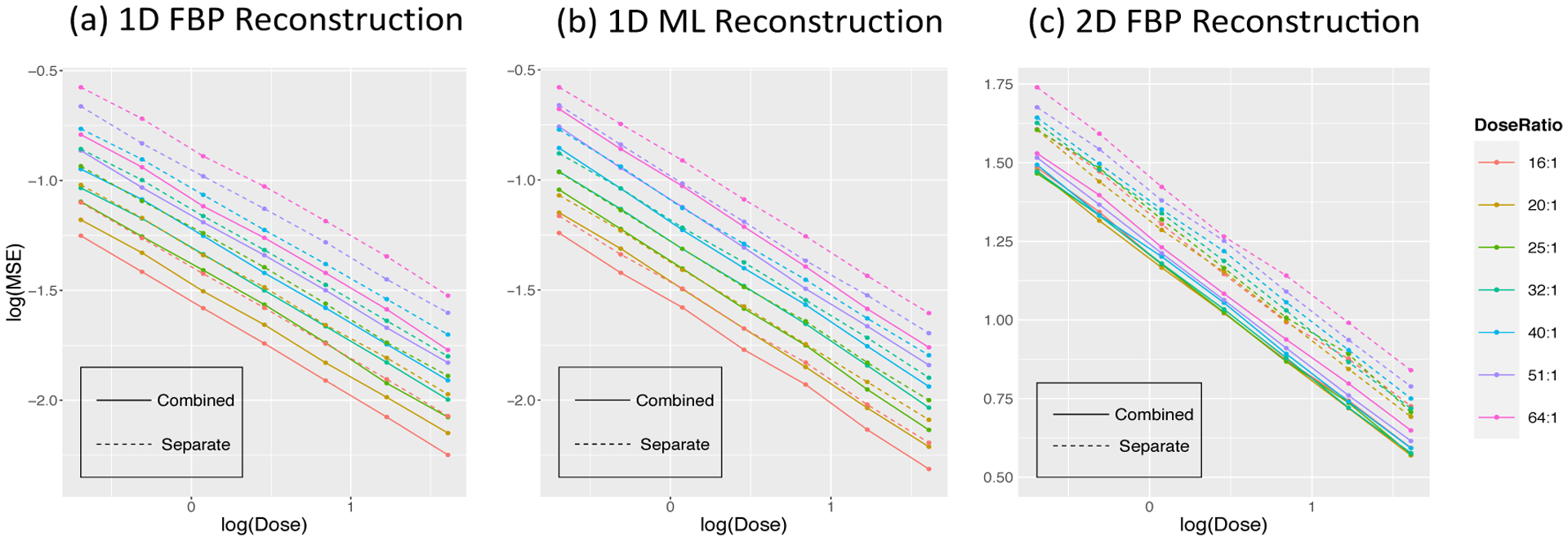
MSE of combined (solid) and separate (dashed) analysis as a function of dose and dose ratio in H_2_O-FDG study. Different colors represent different dose ratios.

The MSE in (14) as the function of the dose and dose ratio from combined and separated approach in 1-D and 2-D simulations are presented in Table 4. Figure 10 presents the information graphically. MSEs from combined and separated analysis decrease with the dose and increase with relative dose ratio. In 1-D simulation studies, the improvements are 20.85% and 11.65% from FBP and ML reconstruction at the reference dose and middle dose-ratio level - matched to the real dataset and improvement is smaller based on the ML reconstructed data. Benefits by combined approach in 2-D simulation with FBP reconstruction is similar to 1D FBP performance.

#### 5.2.2. Repeat H_2_O Studies

**Figure 11:**
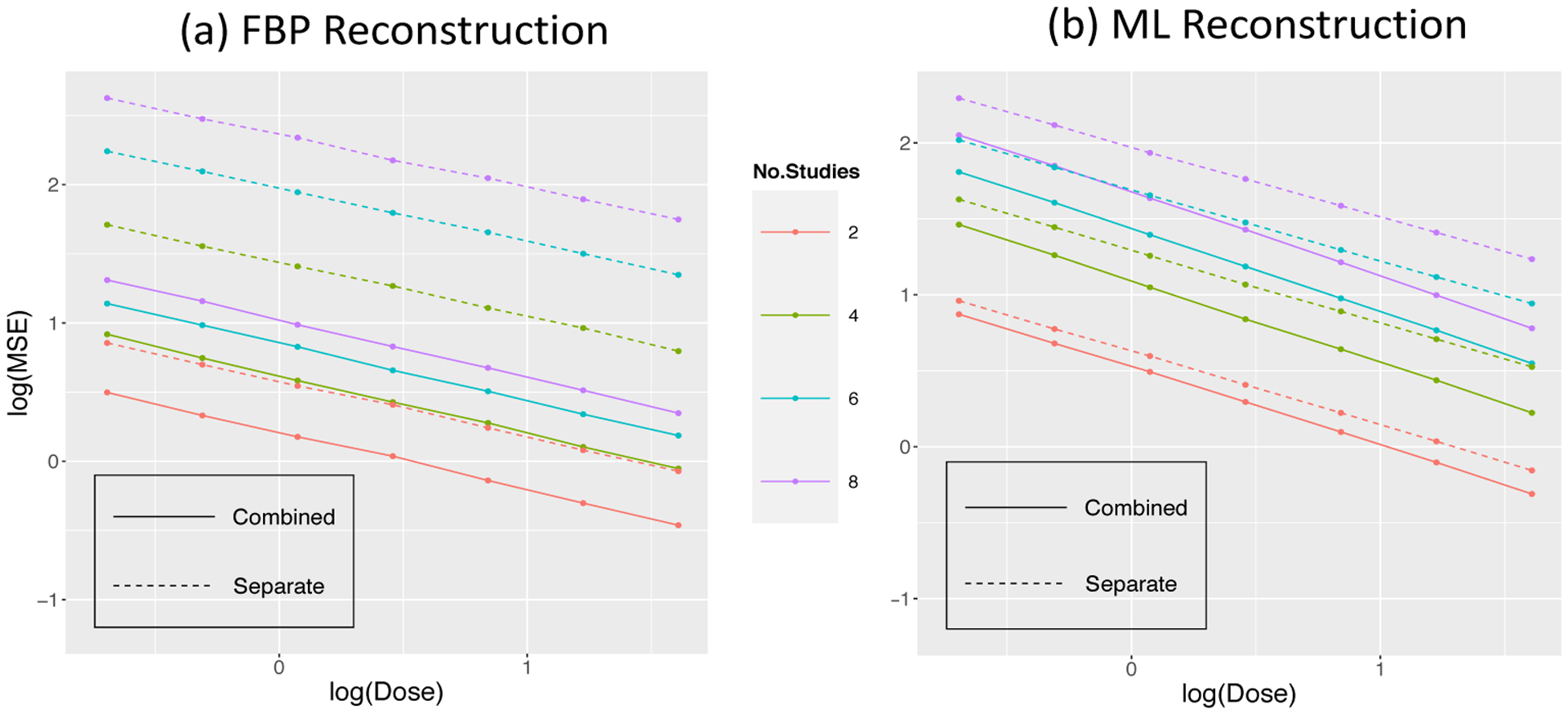
MSE of combined and separate analysis in multiple H_2_O studies.

The quantitative improvements for different numbers of injections at seven dose levels from two reconstruction algorithms are presented in Table 5. We can see, the number of injections has a strong effect. See also Figure 11. These results show lower doses and more injections have higher MSEs. Dramatically smaller improvements are seen with ML. This may be because the bandwidth of smoothing process used in reconstruction is adapted based on the total update scan so there is a more limited impact on individual time frames; the ML procedure constrains the reconstruction to be positive and in doing so implicity smooths the voxel-level time-course data.

## 6. Statistical Analysis of Simulation Results

The highly structured nature of the MSE patterns in Figure 10 and 11, invites a more refined interpretation.

### 6.1. MSE values scaled by Image Data Accuracy

**Figure 12:**
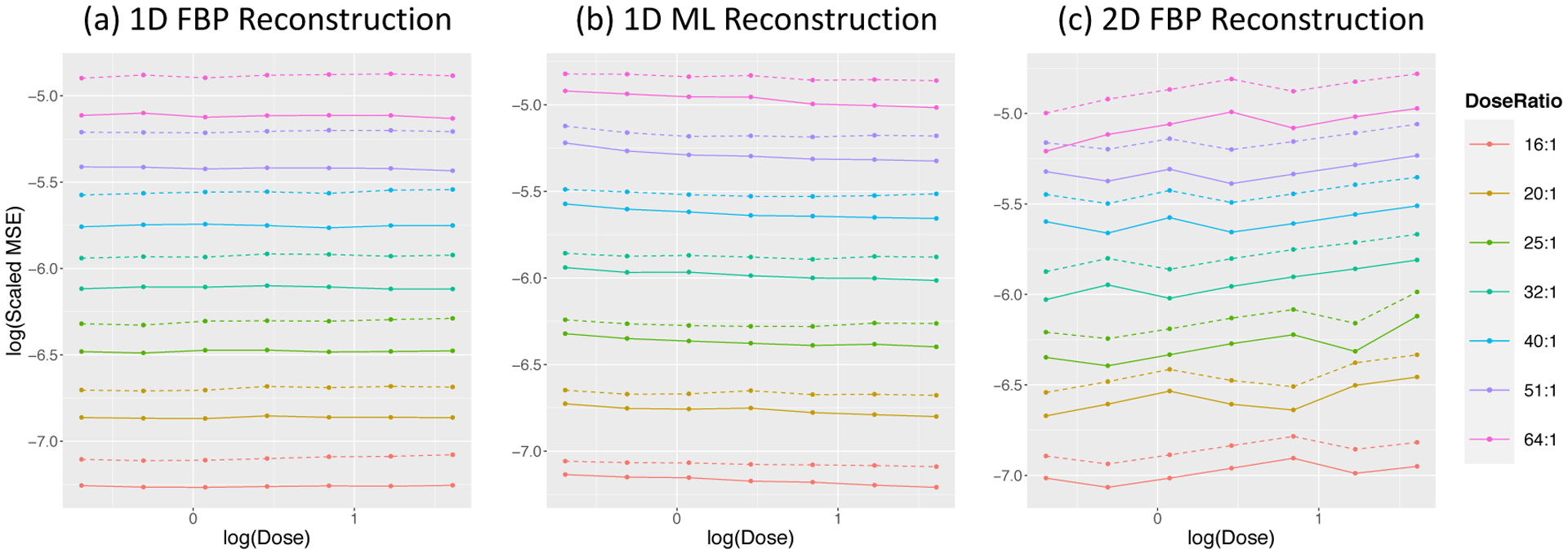
MSE values scaled by image data accuracy for combined (solid) and separate (dashed) analysis as a function of dose and dose ratio in H_2_O-FDG study.

The overall accuracy of the input data is assessed by ${\hat{\sigma }}_{z}^{2}=\frac{1}{N}\underset{i}{\sum }{\hat{\sigma }}_{i}^{2}$ - where ${\hat{\sigma }}_{i}$ was introduced in (7). While we can scale MSE values by this, a simpler (and statistically equivalent) procedure is to scale by the voxel-by-voxel error in the total-uptake reconstruction. We label this ${\hat{\sigma }}_{z•}^{2}$. For the H_2_O-FDG study, the scaled values are in Figure 12.

These data are well described by the model

(16) $$log\left(MSE/{\hat{\sigma }}_{z•}^{2}\right)\sim a_{0}+a_{1}I+a_{2}DR$$

where *I* is the method indicator for combined or separate analysis and *DR* is the dose ratio. Table 6 reports estimates values for these coefficients. Note ${\hat{a}}_{1}$ corresponds to about 21% (*e*^0.19^ − 1) and 12% (*e*^0.11^ − 1) improvements at middle dose-ratio with the FBP and ML reconstruction in 1-D simulation studies. The results from 2D simulation have the similar performance.

A similar analysis of the scaled MSE data from the repeat H_2_O study, leads to a model with an interaction term:

(17) $$log\left(MSE/{\hat{\sigma }}_{z•}^{2}\right)\sim \beta_{0}+\beta_{1}I+\beta_{2}No.\text{Studies}+\beta_{3}\left(I\times No.\text{Studies}\right)$$

**Figure 13:**
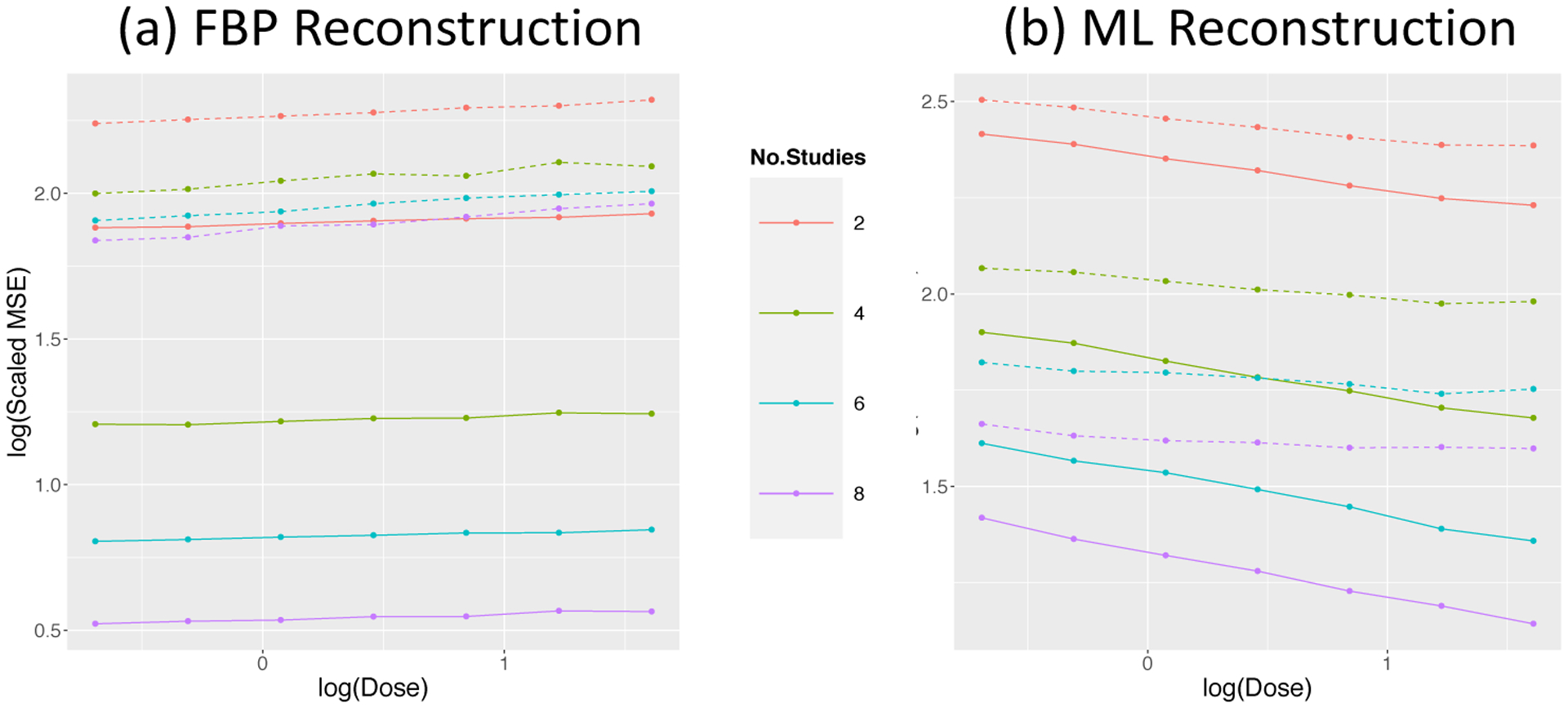
MSE values scaled by image data accuracy for combined (solid) and separate (dashed) analysis as a function of dose and number of studies in the repeat H_2_O setting.

The scaled MSE values are in Figure 13 with the model results in Table 7. The improvements of combined analysis in the repeat H_2_O studies with two injections are 55% and 15% for the FBP and ML reconstructed data, respectively. The impact of dose, although significant, is not substantial in comparison to the overall impact of dose on MSE.

### 6.2. Analysis of the Uptake MSE as a function of Dose

Analysis of MSE of total uptake from FBP and ML reconstruction as a function of dose gives a 2-factor statistical model:

(18) $$log\left({\hat{\sigma }}_{z•}^{2}\right)\sim \gamma_{0}-\gamma_{\tau }log(\tau )+\gamma_{M}M$$

where *τ* is dose level, M is dose ratio (DR) for H_2_O-FDG study and number of injections for repeat H_2_O studies, respectively. The estimated coefficients ${\hat{\gamma }}_{0}$, ${\hat{\gamma }}_{\tau }$ and ${\hat{\gamma }}_{M}$ are shown in Table 8. We can see ${\hat{\gamma }}_{\tau }$ is similar in different studies and reconstruction methods. The intercept - ${\hat{\gamma }}_{0}$ in FBP is always bigger than ML, which means uptake based on ML has smaller errors. Table 8 shows that the main effect of dose is described by *τ*^−.42^ - this is consistent with previous asymptotic analyses of ML and FBP in PET, e.g. (O’Sullivan; 1995). The fact that the rate of convergence is so similar between the 1-D and 2-D simulation models is a good indicator that the 1-D PET framework captures the estimation complexity of 2-D PET.

**Figure 14:**
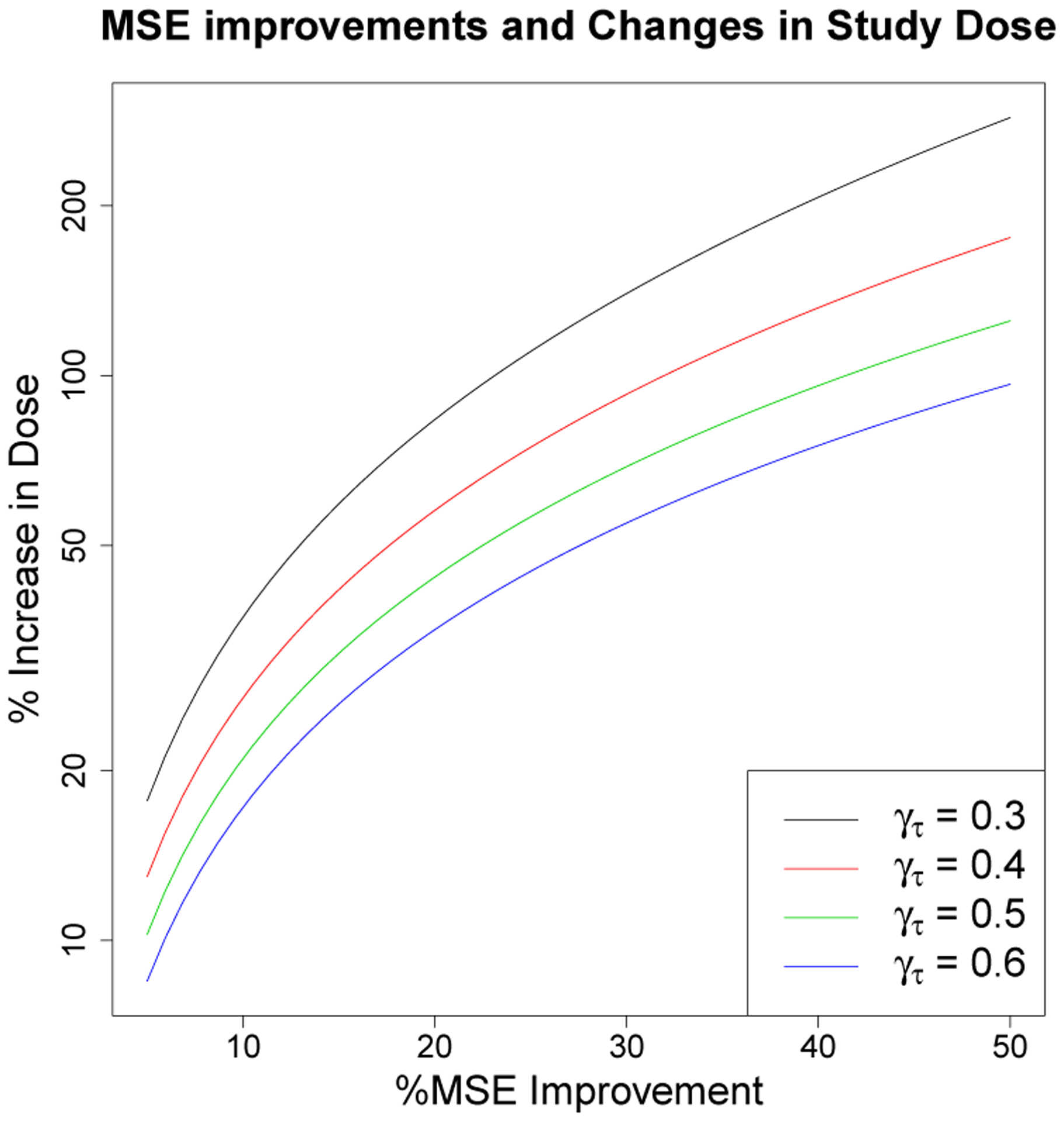
Estimated relation between MSE improvements and study dose. Calculation based on (18) with different colors corresponding to a range of possible *γ*_*τ*_ values.

The rate of convergence analysis allows us to develop a relation between percent improvements obtained by combined data analysis and the equivalent precent increase in dose that would be needed to ensure that the separate data matched the benefits of combined analysis. Figure 14 presents this equivalence. In light of Table 8, which gives a dose rate coefficient on the order of 0.42 for ML and FBP, a 10% improvement in MSE obtained by combined data analysis would require a 25% increase in dose if separate data analysis methods were used.

## 7. Discussion

We have presented a combination of theoretical intuition, real data and numerical simulation to examine the benefits of combined NPRM analysis of multiple injection studies in PET. This work has been fully motivated by practical imaging protocols. The results demonstrate that there are clear gains in MSE performance by a combined analysis approach. With H_2_O-FDG studies the MSE improvement is around 21% on the same order of the regional variance comparisons found in section 3. For the repeat H_2_O studies, improvements increase in accordance with the factor (*J* − 1) indicated by the theory in section 2. In general improvements are seen to be a function of intra-injection dose-ratio but are substantially independent of dose for FBP reconstructed data. In the ML case, the impact of dose is apparent with combined analysis leading to greater improvements at higher doses. Even though the theoretical analysis looks at the asymptotic situation in which variance is the main contributor to the error, the predictions from that theory provide good guidance on the nature of benefits that would be associated with combined analysis.

Our work has exclusively focused on parameter mean square error performance (MSE and RMSE). In kinetic mapping, the average accuracy of estimation of the target parameters, measured by the RMSE, is a natural metric and is used extensively in quantifying estimation error in many settings. Nevertheless, a number of other metrics could also be of interest. Data-fit or predictive error, with suitable adjustment for model flexibility, might also be considered. In addition, criteria based on parameters defined by functions of the kinetics variables associated with individual tracers would also be of interest. MSE improvements associated with combined analysis of ML-reconstructed data are smaller than those achieved with FBP-reconstructed data. This phenomenon may well have to do with the implicit regularization that ML achieves in low-count frames. Our studies used a common bandwidth for all injections. While this gives some assurance that the resolution of each scan is the same for FBP; it is not clear, given the implicit regularisation achieved by imposition of positivity, if this is necessarily the case for ML. The effect is probably most significant in the repeat studies with many injections. In this setting the total uptake bandwidth selection will lead to very noisy individual scans. The bandwidth selection process ensures voxel-level FBP time-course data are inherently noisier than in ML so the analysis of kinetics for FBP data can end up being worse than the same analysis of ML. The issue is apparent in Table 5. An obvious way to address this issue could be to select bandwidths for individual injections separately. This may allow the noise characteristics of FBP to be more comparable to ML (O’Sullivan; 1995; O’Sullivan and Roy Choudhury; 2001). The issue merits further investigation - (Gu and O’Sullivan; 2021) reports on some initial work in this direction.

For computational reasons we made extensive use of a 1-D scanning model in this work. This allowed comprehensive evaluation of estimators with iteratively reconstructed ML data over a range of count rates and many replicates. While FBP is used very little in practice, we included it in simulations. The relation between 1-D and 2-D FBP results might be used as a vehicle to understand how combined analysis methods of 1-D ML data might perform in a 2-D or 3-D setting. Our simulations here demonstrate that combined analysis will significantly improve the accuracy of NPRM kinetic mapping in multiple injection studies. It will be important to see how these results translate into 3-D and especially for the next generation total-body PET scanners (Badawi et al.; 2019; Karp et al.; 2020). In this context the emerging techniques for efficient bootstrapping of 3-D multi-frame PET studies (Kucharczak et al.; 2018; Gu et al.; 2019; Huang et al.; 2020) may facilitate a more direct practical assessment of the benefits of combined analysis in a given patient, without the need for highly sophisticated step-by-step representations of the scanner emission measurements and reconstruction processes.

## Figures and Tables

**Table 1:** Relative Performance (6) of NPRM and 2C kinetic mapping in a PET-FDG setting. Based on a 1-D simulation model with source sub-TACs in case I matched to real Breast Cancer data Figure 8 and to their best-fitting 1C model fit in case II. Data reconstruction by both direct (FBP) and iterative (ML) methods.

Parameters *(p)*		*V*_*B*_	*V*_*D*_	*K*_*D*_	*K*_*i*_	MTT	*K*_*i*_*/K*_1_
I	FBP	1.64 ± 0.02	1.45 ± 0.03	1.13 ± 0.02	1.06 ± 0.01	1.60 ± 0.03	1.60 ± 0.05
	ML	1.78 ± 0.02	1.43 ± 0.03	1.19 ± 0.02	1.05 ± 0.01	1.51 ± 0.03	1.65 ± 0.05
II	FBP	1.32 ± 0.01	1.36 ± 0.02	0.92 ± 0.02	0.97 ± 0.01	0.97 ± 0.01	0.84 ± 0.02
	ML	1.38 ± 0.02	1.31 ± 0.02	0.97 ± 0.03	0.96 ± 0.01	0.94 ± 0.01	0.85 ± 0.02

**Table 2:** Impact of data-adaptive sub-TAC selection in NPRM on Kinetic Mapping errors. Statistical model: ${\text{RMSE}}_{p}^{\text{unknown}}\approx \beta_{p}{\text{ RMSE }}_{p}^{\text{known}}$. Results based on a 2D PET-FDG simulation with FBP reconstruction. Standard errors for estimates are the order of 0.01.

Parameters (*p*)	*V*_*B*_	*V*_*D*_	*K*_*D*_	*K*_*i*_	MTT	*K*_*i*_*/K*_1_
*β*	0.99	0.97	0.82	1.01	0.92	1.03
*R*^2^	0.99	0.97	0.93	0.97	0.90	0.94

**Table 3:** Voxel-level errors in NPRM kinetics as a function of the local reconstruction (${\hat{\sigma }}_{i}$) and modelling (${\hat{\sigma }}_{i}^{\lambda }$) errors - *c.f*. (7),(8). Results based on 1D- PET-FDG simulation.

	Parameters (*p*)	*V*_*B*_	*V*_*D*_	*K*_*D*_	*K*_*i*_	MTT	*K*_*i*_*/K*_1_
FBP	*β*	0.82	1.03	0.82	0.95	0.99	0.97
	*R*^2^	0.96	0.93	0.88	0.99	0.99	0.97
ML	*β*	0.78	1.02	0.88	0.97	0.99	0.98
	*R*^2^	0.78	0.88	0.87	0.98	0.98	0.96
FBP	*β*′	0.86	1.03	0.88	0.97	0.99	0.99
	*R*^2^	0.98	0.92	0.92	0.99	0.98	0.96
ML	*β*′	0.77	1.01	0.89	0.98	0.99	0.99
	*R*^2^	0.83	0.88	0.88	0.98	0.98	0.96

**Table 4:** Combined analysis improvements (15) by dose level/ratio for H_2_O-FDG study.

	_Dose Ratio_╲^Dose^	0.3	0.5	0.7	1	1.5	2.2	3.2
(a) FBP (1D)	16 : 1	16.97	17.12	17.37	18.01	18.71	19.25	19.79
	20 : 1	17.67	17.60	18.55	19.13	19.38	20.10	19.70
	25 : 1	18.13	18.05	18.94	19.09	19.95	20.85	21.17
	32 : 1	20.10	19.78	19.47	20.85	21.35	21.38	22.34
	40 : 1	20.91	20.80	21.16	22.20	22.79	23.44	23.75
	51 : 1	23.06	23.11	23.94	24.30	25.23	25.27	26.07
	64 : 1	25.08	25.93	26.57	27.22	27.26	27.99	28.86
(b) ML (1D)	16 : 1	8.23	8.91	9.19	10.44	10.77	12.34	12.97
	20 : 1	8.39	8.74	9.40	10.81	11.29	12.80	13.38
	25 : 1	8.55	9.08	9.55	10.45	11.84	13.27	14.81
	32 : 1	8.87	10.18	10.38	11.65	11.64	13.76	14.88
	40 : 1	8.98	10.75	10.81	12.11	12.33	13.92	15.65
	51 : 1	10.55	11.42	11.60	12.76	13.92	15.50	16.05
	64 : 1	10.71	12.35	12.69	13.61	15.06	16.70	17.23
(c) FBP (2D)	16 : 1	12.98	13.80	13.73	13.30	12.82	14.21	14.17
	20 : 1	13.83	13.26	12.75	14.02	13.82	13.28	13.08
	25 : 1	14.93	16.23	15.38	15.19	14.89	16.78	14.30
	32 : 1	16.75	15.74	17.37	16.60	16.31	15.65	15.26
	40 : 1	16.21	17.72	16.19	17.85	17.87	17.82	17.10
	51 : 1	17.39	19.27	18.33	20.74	19.71	19.29	18.93
	64 : 1	23.42	21.59	21.16	19.95	22.55	21.33	21.08

**Table 5:** Combined analysis improvements (15) in multiple H_2_O studies at 7 dose levels.

	_No.Studies_╲^Dose^	0.3	0.5	0.7	1	1.5	2.2	3.2
(a) FBP	2	45.69	46.76	46.67	46.54	48.39	48.49	49.48
	4	125.91	129.36	133.47	136.76	133.89	140.95	137.81
	6	208.94	211.81	214.03	219.74	222.75	226.32	225.94
	8	284.02	283.77	297.29	293.94	304.76	306.66	314.65
(b) ML	2	9.51	10.17	11.21	12.14	13.66	15.19	17.15
	4	18.31	20.55	23.44	26.02	28.72	31.48	35.92
	6	23.67	26.53	29.95	33.94	37.92	42.47	48.95
	8	27.86	31.05	35.06	39.93	45.60	51.66	58.28

**Table 6:** Modelling Scaled MSE in the H_2_O-FDG study - *c.f*. (16).

	${\hat{a}}_{0}$	${\hat{a}}_{1}$	${\hat{a}}_{3}$
FBP(1D)	−6.29 ± 0.03	0.19 ± 0.04	1.40 ± 0.04
ML(1D)	−6.17 ± 0.03	0.11 ± 0.04	1.43 ± 0.04
FBP(2D)	−6.10 ± 0.03	0.15 ± 0.04	1.26 ± 0.04

**Table 7:** Modelling Scaled MSE in the repeat H_2_O study, see (17).

	${\hat{\beta }}_{0}$	${\hat{\beta }}_{1}$	${\hat{\beta }}_{2}$	${\hat{\beta }}_{3}$
FBP	1.80 ± 0.03	0.44 ± 0.04	−0.45 ± 0.01	0.32 ± 0.02
ML	2.23 ± 0.03	0.14 ± 0.04	−0.34 ± 0.02	0.07 ± 0.02

**Table 8:** MSE characteristics of reconstructed data in H_2_O-FDG and H_2_O studies (H_2_O*) based on FBP and ML. Analysis is based on equation (18).

		${\hat{\gamma }}_{0}$	${\hat{\gamma }}_{\tau }$	${\hat{\gamma }}_{M}$
FBP(1D)	H_2_O	2.91 ± 0.01	**0.42** ± 0.01	−0.20 ± 0.01
	FDG	4.41 ± 0.02	**0.42** ± 0.03	−1.41 ± 0.04
	H_2_O-FDG	4.75 ± 0.02	**0.42** ± 0.03	−1.10 ± 0.04
	H_2_O*	−1.71 ± 0.05	**0.44** ± 0.04	0.71 ± 0.03
ML(1D)	H_2_O	2.78 ± 0.01	**0.42** ± 0.01	−0.16 ± 0.01
	FDG	4.30 ± 0.02	**0.43** ± 0.03	−1.40 ± 0.04
	H_2_O-FDG	4.65 ± 0.02	**0.43** ± 0.03	−1.06 ± 0.04
	H_2_O*	−1.85 ± 0.05	**0.43** ± 0.04	0.72 ± 0.03
FBP(2D)	H_2_O	−2.21 ± 0.01	**0.44** ± 0.01	0.03 ± 0.02
	FDG	−2.15 ± 0.01	**0.37** ± 0.01	−0.03 ± 0.01
	H_2_O-FDG	−2.33 ± 0.01	**0.42** ± 0.01	−0.05 ± 0.02

## Appendix: Derivation of Formula in (11)

Gaussian approximation of estimates of *α*-coefficients means that as any voxel we can assume

(19) $${\hat{\alpha }}^{j}\sim N\left(\alpha ,\Sigma_{j}\right)$$

where Σ_*j*_ is a covariance matrix associated with the weighted least squares estimation process. Within the Gaussian framework ${\hat{\alpha }}^{j}$ is a sufficient statistic, *i.e*. it captures the full information about *α* based on the *j*’th can data. As a result the optimal (maximum-likelihood) combined estimator of *α* is a matrix-weighted average of the individual scan estimators, *i.e*.

(20) $${\hat{\alpha }}^{C}={\left[\underset{j^{*}}{\sum }\sum_{j^{*}}^{-1}\right]}^{-1}\left[\underset{j}{\sum }\sum_{j}^{-1}{\hat{\alpha }}^{j}\right]\;;\;{\hat{\alpha }}^{C}\sim N\left(\alpha ,\sum_{C}\right)$$

where $\sum_{C}={\left[\sum_{1}^{-1}+\ldots +\sum_{J}^{-1}\right]}^{-1}$. We can write ∑_*C*_ as $\sum_{j}^{1/2}{\left[I+B_{j}\right]}^{-1}\sum_{j}^{1/2}$. where

(21) $$B_{j}=\underset{j^{*}\neq j}{\sum }\sum_{j}^{1/2}\sum_{j^{*}}^{-1}\sum_{j}^{1/2}$$

Clearly *B*_*j*_ is symmetric and positive definite. Thus if *λ*_*j*_ is the smallest eigenvalue of *B*_*j*_, then for any vector *v*, we have

(22) $$v^{\prime }{\left[I+B_{j}\right]}^{-1}v\leq {\left[1+\lambda_{j}\right]}^{-1}v^{\prime }v$$

Since *λ*_*j*_ ≥ 0, it follows that for any parameter (*θ*) defined by a linear combination *θ* = *ξ*′*α*

(23) $$\frac{E{\left[{\hat{\theta }}^{j}-\theta \right]}^{2}}{E{\left[{\hat{\theta }}^{C}-\theta \right]}^{2}}=\frac{Var\left({\hat{\theta }}^{j}\right)}{Var\left({\hat{\theta }}^{C}\right)}\geq \left[1+\lambda_{j}\right]\geq 1$$

*i.e*. the combined estimator will always have a smaller MSE than the single scan estimate.

More generally suppose that Gaussian approximation applies directly to the kinetic parameter, *θ* = *g*(*α*). This might follow by first-order Taylor series expansion of $g(\hat{\alpha })$ about *α* (Van der Vaart; 2000). With this we would have

(24) $${\hat{\theta }}^{j}\sim N\left(\theta ,\sigma_{j}^{2}\right)\text{ where }\sigma_{j}^{2}=v^{\prime }\sum_{j}v$$

and *v* is the gradient of *g* evaluated at *α*. As in (19), ${\hat{\theta }}^{j}$ is now (to within the approximation) sufficient for estimation of *θ* based on the information acquired in the *j*’th scan. The optimal combined scan estimator is a weighted average of these estimates

(25) $${\hat{\theta }}_{C}=\frac{\sigma_{1}^{-2}{\hat{\theta }}_{1}+\sigma_{2}^{-2}{\hat{\theta }}_{2}+\ldots +\sigma_{J}^{-2}{\hat{\theta }}_{J}}{\sigma_{1}^{-2}+\sigma_{2}^{-2}+\ldots +\sigma_{J}^{-2}}$$

Thus $\theta^{C}\sim N\left(\theta ,\sigma_{C}^{2}\right)$ with $\sigma_{C}^{2}=\frac{1}{\sigma_{1}^{-2}+\sigma_{2}^{-2}+\ldots +\sigma_{J}^{-2}}$. The result in (11) follows from this.
